# Placebo effects on cutaneous pain and itch: a systematic review and meta-analysis of experimental results and methodology

**DOI:** 10.1097/j.pain.0000000000002820

**Published:** 2022-11-16

**Authors:** Joseph S. Blythe, Mia A. Thomaidou, Kaya J. Peerdeman, Antoinette I.M. van Laarhoven, Myrthe M.E. van Schothorst, Dieuwke S. Veldhuijzen, Andrea W.M. Evers

**Affiliations:** a Health, Medical and Neuropsychology Unit, Leiden University, Leiden, the Netherlands; b Leiden Institute for Brain and Cognition, Leiden, the Netherlands; c Medical Delta Healthy Society, Leiden University, Technical University Delft, and Erasmus University Rotterdam, Rotterdam, the Netherlands; d Department of Psychiatry, Leiden University Medical Centre, Leiden, the Netherlands

**Keywords:** Placebo effects, Pain, Itch, Conditioning, Verbal suggestion, Observational learning

## Abstract

Placebo effects, positive treatment outcomes that go beyond treatment processes, can alter sensations through learning mechanisms. Understanding how methodological factors contribute to the magnitude of placebo effects will help define the mechanisms by which these effects occur. We conducted a systematic review and meta-analysis of experimental placebo studies in cutaneous pain and itch in healthy samples, focused on how differences in methodology contribute to the resulting placebo effect magnitude. We conducted meta-analyses by learning mechanism and sensation, namely, for classical conditioning with verbal suggestion, verbal suggestion alone, and observational learning, separately for pain and itch. We conducted subgroup analyses and meta-regression on the type of sensory stimuli, placebo treatment, number of acquisition and evocation trials, differences in calibrated intensities for placebo and control stimuli during acquisition, age, and sex. We replicated findings showing that a combination of classical conditioning with verbal suggestion induced larger placebo effects on pain (*k* = 68, *g =* 0*.*59) than verbal suggestion alone (*k* = 39, *g =* 0.38) and found a smaller effect for itch with verbal suggestion alone (*k* = 7, *g* = 0.14). Using sham electrodes as placebo treatments corresponded with larger placebo effects on pain than when topical gels were used. Other methodological and demographic factors did not significantly affect placebo magnitudes. Placebo effects on pain and itch reliably occur in experimental settings with varied methods, and conditioning with verbal suggestion produced the strongest effects. Although methods may shape the placebo effect to some extent, these effects appear robust overall, and their underlying learning mechanisms may be harnessed for applications outside the laboratory.

## Abstract 2

Supplemental Digital Content is Available in the Text.

## 1. Introduction

Placebo effects, positive treatment outcomes for sensations such as pain and itch that arise through psychobiological mechanisms independent of an actual treatment,^53,56^ are routinely observed in clinical trials and practice.^85,142^ Their prevalence and magnitude likely vary across conditions and contexts, but these effects are thought to occur in many clinical trial participants receiving placebos,^46^ and magnitudes can vary extensively, from no effect to large effects.^141^ Placebo effects are routinely studied in healthy participants,^15,32^ allowing for better-controlled investigation of the underlying mechanisms compared with research in clinical settings. Although these effects are most often studied in pain, itch is a similar but distinct sensation with overlapping neurobiological mechanisms,^122^ highly susceptible to psychological influence.^2,100,126^ Placebo effects on itch routinely occur in the treatment of dermatological conditions,^139^ but their relation to placebo effects on pain is not well understood. A deeper investigation of the factors that shape the magnitude of both placebo effects on pain and itch will further our understanding of when and how these effects occur, and the mechanisms that underlie them.

In mechanistic placebo research, positive treatment expectations are typically induced using classical conditioning, verbal suggestions, observational learning, or a combination of these learning processes.^28^ Classical conditioning induces placebo effects by forming associations between an (inert) treatment and a decrease in sensation^8,13^; initially reinforced with a genuine reduction in sensation, the effect of which becomes associated with the inert treatment. For example, if one experiences pain relief every time they take a given medicine, they may come to expect pain relief from this medicine. Those expectations alone may be enough to foment some pain relief, such that if this person ingested a pill that they believed to be their analgesic medicine but was in fact a placebo, they would still experience some degree of pain reduction. Verbal suggestions explicitly provide positive information regarding the pain-relieving or itch-relieving effects of a treatment.^140^ This could come in the form of a doctor telling you that a new medicine will reduce your itch symptoms, inducing expectations for this outcome, which propagate some degree of itch relief on top of any biological effects of the treatment. Placebo effects can also be formed by observing the effects of a pain-relieving or itch-relieving treatment in another person.^10,131^ Observational learning could, for example, form expectations for pain relief by seeing a friend's pain symptoms improve after trying a different physical therapy exercise. There seems to be an additive benefit to combining multiple learning processes when inducing these effects,^12,28^ although this has not been systematically reviewed.

One goal of experimental research into placebo effects on pain and itch has been to identify factors that influence these effects.^98,143^ Methods used in experimental placebo research are heterogeneous, varying factors such as the type of sensation (eg, thermal pain, electrical pain), the type of placebo intervention (eg, sham electrodes, gels, or pills), the number of acquisition and evocation trials used in a conditioning design,^33,124^ and the difference in intensity of pain stimulations between placebo and control trials.^63^ Demographic characteristics of study populations like sex and age may also potentially impact resulting placebo effects. Although some studies investigating sex differences in placebo responses have found that men are more responsive to verbal suggestions for placebo effects on pain,^52,138^ findings are mixed for classical conditioning and remain unexplored for itch. Age differences across the adult lifespan similarly have not been investigated for placebo effects on pain or itch. Systematic review and meta-analysis allows us to study what influence these methodological and demographic factors may have across studies. Previous meta-analyses of placebo effects on pain and itch have documented their widespread prevalence in clinical trials,^139,142^ and for pain, they demonstrated that mechanistic research tends to find larger placebo effects than those seen in clinical research. The use of longer pain stimuli was also associated with larger placebo effects.^141^ Since the most recent meta-analysis of mechanistic research into placebo effects on pain over a decade ago,^141^ numerous new studies have been published, particularly studies with healthy samples. To date, no meta-analyses have sought to quantify the magnitude of experimentally induced placebo effects on itch, nor have methodological factors been studied systematically as a potential source of heterogeneity in placebo effect magnitudes for pain or itch.

Given the growing body of research into placebo effects in cutaneous sensations, a systematic review and meta-analysis is warranted to provide insights into the distinct contributions of experimental components. Examining placebo effects across the literature may provide a better understanding of how these effects can be enhanced and potentially used in clinical settings, creating research avenues for novel therapies or informing doctor–patient communication. In pursuit of this aim and building on previous meta-analyses of similar scope,^110,111,141,142^ we conducted a systematic review and meta-analysis on the magnitude of placebo effects for inert treatments, in experiments on pain and itch, in healthy participants. First, we assessed the magnitude of placebo effects (defined as the decrease in pain or itch intensity after an inert treatment compared with a within-subject or between-subject control) by learning process (verbal suggestion, classical conditioning with verbal suggestion, and observational learning). To investigate the role of methodological and demographic factors, we then conducted subgroup analyses assessing the effect of the type of cutaneous sensation and the type of placebo intervention, and meta-regression to assess the impact of the number of learning and evocation trials used in classical conditioning models, the difference in calibrated intensity of placebo and control stimulations, sex distribution, and mean age of the participants on placebo-effect magnitudes.

## 2. Methods

### 2.1. Protocol and registration

The protocol for this study was preregistered on ClinicalTrials.gov (ID: NCT04387851) and was conducted following PRISMA guidelines^106^ (Supplementary digital content, available at http://links.lww.com/PAIN/B752). We registered a single search strategy for placebo and nocebo studies, the results of which have been divided into 2 articles after evaluating the amount of articles yielded by the search, facilitating a more clear and nuanced discussion for each set of findings. Here, we report on the placebo studies.

### 2.2. Databases and selection criteria

PubMed, PsycINFO, EMBASE, and the Cochrane CENTRAL Methodology Library were searched to identify studies. Languages were limited to English, Dutch, and German, and the publication period was not restricted. Searches were initially conducted on March 18, 2019, and subsequently updated on April 10, 2020, and July 15, 2021. The complete key-worded search strategy for each database is available in the supplementary digital content (available at http://links.lww.com/PAIN/B752).

We searched for original, controlled experimental studies on healthy participants that aimed to experimentally induce placebo or nocebo effects on cutaneous sensations (ie, pain or itch stimulations that were administered on the skin); of which, the results of studies on placebo effects are reported here. Patient samples were not included because the current review focuses on learning mechanisms and methodological factors, which can be studied with better experimental control in healthy samples. For better homogeneity of study designs, we focused only on cutaneous sensations, and excluded, eg, studies on visceral or ischemic sensation. For the purposes of inclusion and exclusion, studies were considered to have induced a placebo effect if a learning mechanism (eg, conditioning, verbal suggestion, observational learning) was used to induce positive expectations about an inert treatment and not to purely ambiguous stimuli (eg, colored shapes). This was done to focus the scope of this review on experimental studies, which induced expectations around treatments as opposed to abstract stimuli, thereby improving the clinical relevance of the meta-analyses. We only included studies that featured some form of control comparator, whether that was within or between subjects, so that the placebo effect could be calculated as the difference between placebo and control. Studies that excluded nonresponders from the analyses were excluded. Studies that did not fulfill one or more of the criteria mentioned above were excluded from further review and meta-analysis. Our search terms did not include words specifically intended to collect observational learning studies because we did not originally plan to investigate this learning mechanism in our preregistration. Still, our search identified observational learning studies, and we decided to include them in our review because we likely identified all relevant observational learning studies with reference list and Web of Science searches.

### 2.3. Study selection

Titles and abstracts of articles retrieved using the above search strategy were independently screened by 2 authors (J.S.B. and M.M.E.V.S). The full text of articles to be included and articles about which doubts existed were then retrieved and assessed for eligibility by 2 authors independently (J.S.B. and M.A.T.). The reference lists of all included articles were also screened for study inclusion by one author (J.S.B.) and a student assistant, and included articles were also entered in Web of Science to identify articles that have cited them and should potentially be included in the meta-analysis in April, 2020. When full texts were not available online, authors were contracted through email to request access. Disagreements concerning study inclusion decisions were resolved by a third author (K.J.P).

### 2.4. Data extraction

One author (J.S.B.) used a standardized form to independently extract data from the included studies to derive study characteristics and data for analyses. Another author (M.A.T.) checked 25% of extracted values for accuracy. Extracted information included details of the experimental induction (ie, learning mechanism used), control condition, study population, placebo treatment, sensation type, pain/itch outcome data, how sensations were measured (eg, 0-10 numeric rating scale, visual analogue scale, 0-20 Gracely scale, etc), type of cutaneous stimulation (eg, heat pain, pressure pain, histamine-evoked itch), information for quality and bias assessment, and outcome data for meta-analysis (eg, sample size, pain/itch rating means and standard deviations). Doubts regarding data extraction were resolved through discussion with a third author (K.J.P.). Missing data were requested from the authors of included studies. If the authors did not respond, but data could be extracted from published figures, this was done with the software WebPlotDigitizer version 4.4 (Rohatgi, 2020).

### 2.5. Risk of bias

#### 2.5.1. Risk of bias assessment within studies

Risk of bias was assessed by a student assistant and one of the authors (M.A.T.), independently from one another, using the method developed by Marcuzzi et al.,^91^ specifically for quantitative sensory testing studies. This method assesses (1) whether the inclusion criteria were clearly described (3 items), (2) whether the sample is clearly described and representative of the population (5 items), (3) whether the recruitment process was clearly described (3 items) (4) whether the somatosensory assessment methods are standardized, validated, and well described (6 items), (5) adequate blinding if relevant (1 item), and (6) whether potential confounders were considered (2 items). Items were scored as satisfied (0 points), not satisfied (2 points), partially satisfied or unclear (1 point), or not applicable. Studies receive a score ranging from 0 to 40 based on these criteria, with higher scores indicating a greater risk of bias. Meta-regression was used to test for a relationship between risk of bias score and the magnitude of the placebo effect. An example of the risk of bias tool can be found in the supplementary digital content (available at http://links.lww.com/PAIN/B752).

#### 2.5.2. Risk of publication bias across studies

Risk of publication bias across studies was assessed visually with funnel plots. Studies lying outside the funnel of expected results were included in subsequent analyses, but their outlier status was noted in the study characteristics table (Tables 1–4). Publication bias was assessed with Duval and Tweedie's trim and fill method,^47^ a nonparametric technique for estimating the number of missing studies in a meta-analysis and the impact these studies would likely have on the overall effect size.

**Table 1:** Characteristics of studies included in the classical conditioning with verbal suggestion (pain) meta-analysis.

Author	Year	N	Sample age	Percent female	Sensation induction method	Placebo manipulation	Rating scale	Acquisition trials (Placebo/Control)	Evocation trials (Placebo/Control)	Calibrated stimulus intensity difference (0-100)*	First or mean outcome measure	Risk of bias score	Comparison	Outlier based on funnel plot
Au Yeung^7^	2014	20	19.8	59%	Electrical	Sham TENS	0-100 VAS	32 (16P/16C)	32 (16P/16C)	NA	First	3	W	
Barnes^11^	2021	62	19.4	52%	Electrical	Sham TENS	0-10 pain intensity	30 (15P/15C)	20 (10P/10C)	45	Mean	3	W	
Case^21^	2019	28	NR	53%	Thermal	Inert gel	0-80 pain intensity	8 (4P/4C)	16 (8P/8C)	NA	Mean	7	W	−
Choi^23^	2011	15	25.3	0%	Electrical	Sham IV	0-100 NRS	Unknown	10 (5P/5C)	NA	Mean	3	W	
Chouchou^24^	2015	26	23.4	46%	Thermal	Inert gel	0-100 VAS	16 (8P/8C)	10 (5P/5C)	35	Mean	4	W	
Colagiuri^27^	2018	21	20.2	71%	Electrical	Sham TENS	0-100 VAS	32 (16P/16C)	32 (16P/16C)	NA	First	5	W	
Colloca^30^	2006	10	22.7	83%	Electrical	Sham electrode	0-10 NRS	36 (18P/18C)	12 (6P/6C)	NA	Mean	5	W	+
Colloca^34^	2008	15	22.5	100%	Electrical	Sham electrode	0-10 VAS	24 (12P/12C)	12 (6P/6C)	NA	Mean	3	W	
Colloca^35^	2008a	16	32.0	66%	Laser	Inert gel	0-10 NRS	30 (15P/15C)	30 (15P/15C)	NA	Mean	5	W	
Colloca^31^	2009	16	22.6	100%	Electrical	Sham electrode	0-10 NRS	24 (12P/12C)	12 (6P/6C)	NA	Mean	5	W	
Colloca^33^	2010	46	22.8	65%	Electrical	Sham electrode	0-10 VAS	20 (10P/10C)	40 (20P/20C)	30	Mean	3	W	+
Colloca^33^	2010	46	22.8	65%	Electrical	Sham electrode	0-10 VAS	80 (40P/40C)	40 (20P/20C)	30	Mean	3	W	+
Colloca^36^	2019	53	28.1	64%	Electrical	Sham electrode	0-10 NRS	18 (9P/9C)	36 (18P/18C)	60	Mean	3	W	
Colloca^29^	2020	400	29.4	59%	Thermal	Sham electrode	0-100 VAS	24 (12P/12C)	12 (6P/6C)	NA	Mean	5	W	
Corsi^37^	2017	46	27.4	52%	Thermal	Sham electrode	0-100 VAS	12 (6P/6C)	6 (3P/3C)	NA	Mean	3	W	
de Jong^39^	1996	36	21.3	100%	Electrical	Inert gel	0-100 VAS	20 (10P/10C)	10 (5P/5C)	25	Mean	5	B	
De Pascalis^40^	2002	36	25.4	65%	Electrical	Inert gel	0-10 VAS	12 (6P/6C)	30 (15P/15C)	NA	Mean	2	W	
De Pascalis^43^	2021	56	23.3	100%	Cold cup	Inert gel	0-100 NRS	2 (1P/1C)	2 (1P/1C)	NA	Mean	4	W	
Egorova^48^	2020	24	NR	50%	Thermal	Inert gel	0-20 Gracely scale	48 (24P/24C)	24 (12P/12C)	25	Mean	5	W	
Eippert^49^	2009	19	25.0	0%	Thermal	Inert gel	0-100 VAS	12 (6P/6C)	30 (15P/15C)	40	Mean	6	W	
Eippert^50^	2009a	13	25.0	0%	Thermal	Inert gel	0-100 VAS	12 (6P/6C)	30 (15P/15C)	40	Mean	0	W	
Feldhaus^55^	2021	624	24.6	60%	Thermal	Inert gel	0-100 VAS	16 (8P/8C)	16 (8P/8C)	40	Mean	3	W	
Flaten^57^	2018	25	21.9	56%	Thermal	Inert pill	0-10 NRS	3 (2P/1C)	2 (1P/1C)	NA	Mean	0	W	
Frangos^58^	2021	46	39.7	85%	Thermal	Inert gel	0-200 VAS	24 (12P/12C)	20 (10P/10C)	45	Mean	3	W	
Freeman^59^	2015	24	NR	50%	Thermal	Inert gel	0-20 Gracely scale	18 (9P/9C)	Unknown	25	Mean	5	W	
Gaab^60^	2019	81	25.2	60%	Thermal	Inert gel	0-10 VAS	16 (8P/8C)	4 (2P/2C)	30	Mean	3	W	
Geisler^62^	2020	33	27.4	0%	Thermal	Inert gel	0-100 VAS	16 (8P/8C)	8 (4P/4C)	40	Mean	3	W	
Geuter^63^	2013	40	26.0	0%	Thermal	Inert gel	0-100 VAS	24 (12P/12C)	30 (15P/15C)	50	Mean	3	W	
Geuter^63^	2013	40	26.0	0%	Thermal	Inert gel	0-100 VAS	24 (12P/12C)	30 (15P/15C)	30	Mean	3	W	
Grahl^66^	2018	23	24.6	0%	Thermal	Sham TENS	0-100 VAS	24 (12P/12C)	24 (12P/12C)	40	Mean	0	W	
Hartmann^67^	2021	45	23.8	51%	Electrical	Inert gel	0-8 pain intensity	Unknown	32 (16P/16C)	30	Mean	1	W	
Huneke^74^	2013	73	37.6	66%	Laser	Inert gel	0-10 NRS	20 (10P/10C)	20 (10P/10C)	40	Mean	3	B	
Jarcho^77^	2016	15	24.3	100%	Thermal	Inert gel	0-100 VAS	2 (1P/1C)	2 (1P/1C)	NA	Mean	4	W	
Kirsch^79^	2014	48	26.4	50%	Thermal	Sham acupuncture	0-20 Gracely scale	Unknown	Unknown	40	Mean	5	B	
Klinger^80^	2007	12	26.1	50%	Electrical	Inert gel	0-8 pain intensity	10 (5P/5C)	10 (5P/5C)	25	Mean	8	W	
Kong^82^	2006	16	28.4	44%	Thermal	Sham acupuncture	0-20 Gracely scale	48 (24P/24C)	24 (12P/12C)	40	Mean	5	W	
Laverdure-Dupont^85^	2009	38	23.4	58%	Thermal	Inert gel	0-100 VAS	16 (8P/8C)	10 (5P/5C)	20	Mean	3	W	
Lee^86^	2020	21	23.6	43%	Pressure	Inert gel	0-100 VAS	12 (6P/6C)	12 (6P/6C)	NA	Mean	5	W	
Lui^89^	2010	31	23.5	58%	Laser	Sham electrode	0-100 VAS	24 (12P/12C)	12 (6P/6C)	NA	Mean	7	W	
Martin^95^	2010	40	21.2	70%	Thermal	Inert gel	0-10 NRS	16 (8P/8C)	2 (1P/1C)	30	Mean	5	W	
Martini^96^	2015	28	23.5	50%	Laser	Inert gel	0-100 NRS	24 (12P/12C)	32 (16P/16C)	NA	Mean	7	W	
Martin-Pichora^94^	2011	15	22.8	68%	Thermal	Inert gel	0-10 NRS	16 (8P/8C)	2 (1P/1C)	30	Mean	3	W	
Montgomery^104^	1997	24	NR	50%	Electrical	Inert gel	0-10 VAS	20 (10P/10C)	12 (6P/6C)	30	Mean	9	B	
Morton^106^	2009	66	25.0	64%	Laser	Inert gel	0-10 pain intensity	60 (30P/30C)	60 (30P/30C)	40	Mean	5	B	
Morton^105^	2010	56	25.0	62%	Laser	Inert gel	0-10 pain intensity	60 (30P/30C)	60 (30P/30C)	40	Mean	7	B	
Power^117^	2020	57	49.0	65%	Laser	Inert gel	0-10 NRS	20 (10P/10C)	20 (10P/10C)	40	Mean	2	W	
Price^118^	1999	34	19.3	60%	Thermal	Inert gel	0-10 VAS	30 (15P/15C)	4 (2P/2C)	40	Mean	6	W	
Rhudy^120^	2018	33	36.4	51%	Electrical	Inert gel	0-100 VAS	24 (12P/12C)	24 (12P/12C)	NA	Mean	6	W	+
Rosén^124^	2016	36	25.0	58%	Thermal	Sham electrode	0-100 NRS	3 (1P/2C)	3 (1P/2C)	NA	Mean	0	W	
Rütgen^126^	2015	102	26.2	67%	Electrical	Inert pill	0-7 pain intensity	4 (2P/2C)	Unknown	25	Unknown	3	B	
Schafer^127^	2015	40	NR	67%	Thermal	Inert cream	0-100 VAS	16 (8P/8C)	40 (24P/16C)	40	Mean	6	W	
Schafer^127^	2015	40	NR	67%	Thermal	Inert cream	0-100 VAS	112 (56P/56C)	40 (24P/16C)	40	Mean	6	W	
Schenk^129^	2017	24	25.4	48%	Thermal	Sham TENS	0-100 VAS	18 (9P/9C)	18 (9P/9C)	40	Mean	3	W	
Skvortsova^131^	2020	37	23.1	0%	Thermal	Inert nasal spray	0-10 NRS	24 (12P/12C)	20 (10P/10C)	30	Mean	0	W	
Tang^138^	2019	30	22.5	67%	Electrical	Sham TENS	0-100 graphic rating scale	32 (16P/16C)	4 (2P/2C)	NA	Unknown	4	W	
Tu^139^	2021	27	27.4	46%	Thermal	Inert cream	0-20 Gracely scale	48 (24P/24C)	24 (12P/12C)	25	Mean	3	W	
Valentini^141^	2014	27	24.9	54%	Laser	Sham electrode	0-100 VAS	24 (12P/12C)	8 (4P/4C)	42	Mean	5	W	
Vambheim^142^	2021	59	21.5	44%	Thermal	Inert gel	0-10 NRS	30 (15P/15C)	30 (15P/15C)	NA	Mean	0	W	−
Vambheim^142^	2021	32	NR	54%	Electrical	Inert gel	0-10 NRS	22 (11P/11C)	36 (18P/18C)	20	Mean	0	W	
Wager^152^	2004	24	NR	NR	Thermal	Inert gel	0-10 VAS	60 (12P/12C)	12 (6P/6C)	60	Mean	12	W	
Wager^149^	2006	39	23.2	55%	Laser	Inert gel	−2 to 10 VAS	10 (5P/5C)	80 (40P/40C)	NA	Mean	7	W	
Wager^150^	2007	15	NR	0%	Thermal	Inert gel	0-10 VAS	10 (5P/5C)	60 (30P/30C)	NA	Mean	7	W	
Watson^154^	2006	24	23.8	55%	Laser	Inert gel	0-100 NRS	20 (10P/10C)	20 (10P/10C)	NA	Mean	7	W	
Watson^156^	2007	18	NR	45%	Laser	Inert gel	0-10 pain intensity	80 (40P/40C)	40 (20P/20C)	40	Mean	7	B	
Watson^155^	2009	11	NR	54%	Laser	Inert gel	0-10 NRS	30 (15P/15C)	30 (15P/15C)	50	Mean	9	W	
Wei^157^	2018	18	20.9	100%	Electrical	Sham electrode	0-10 pain intensity	40 (20P/20C)	16 (8P/8C)	NA	Mean	4	W	
Weimer^159^	2019	78	27.5	73%	Thermal	Inert gel	0-10 VAS	16 (8P/8C)	16 (8P/8C)	30	Mean	2	W	
Weng^160^	2021	32	22.0	75%	Thermal	Sham electrode	0-10 NRS	30 (15P/15C)	10 (5P/5C)	25	Mean	1	W	
Wrobel^162^	2014	17	26.6	46%	Thermal	Inert gel	0-100 NRS	36 (18P/18C)	30 (15P/15C)	40	Mean	0	W	
Wrobel^161^	2015	23	27.5	37%	Thermal	Inert gel	0-100 VAS	30 (15P/15C)	24 (12P/12C)	30	Mean	7	W	
Zunhammer^163^	2018	33	25.0	50%	Thermal	Inert gel	0-100 VAS	30 (15P/15C)	30 (15P/15C)	40	Mean	1	W	

For studies using a within-subject comparison to measure the placebo effect, N is reported as the number of participants from the group in which the comparison was made. For studies using a between-subject comparison, N is reported as the combined number of participants from the placebo and control groups from which the comparison was made, or only the placebo condition in the case of within subjects comparisons.

* Instead of using calibrated stimulus intensities, some studies used fixed intensities, which were recorded here as NA. In the final column, “+” indicates a potential outlier with an effect size larger than the bounds of a 99% confidence interval, and “−” indicates a potential outlier with an effect size smaller than the bounds of a 99% confidence interval, based on inspection of funnel plots (Supplementary Materials, http://links.lww.com/PAIN/B752).

C, control; IV, intravenous; N, sample size; NA, not applicable; NR, not reported; NRS, numeric rating scale; P, placebo; TENS, transcutaneous electric nerve stimulation; VAS, visual analogue scale.

**Table 2:** Characteristics of studies included in the verbal suggestion (pain) meta-analysis.

Author	Year	N	Sample age	Percent female	Sensation induction method	Placebo manipulation	Rating scale	Risk of bias score	Comparison	Outlier based on funnel plot
Aslaksen^4^	2008	63	24.2	51%	Thermal	Inert pill	0-100 VAS	0	W	
Aslaksen^6^	2015	48	23.4	51%	Thermal	Inert gel	0-100 VAS	3	B	
Aslaksen^3^	2016	32	21.6	54%	Thermal	Inert gel	0-100 VAS	3	B	
Brown^17^	2013	61	19.5	59%	Cold pressor	Inert gel	0-10 NRS	5	W	
Camerone^19^	2021	21	22.0	52%	Electrical	Inert gel	0-10 NRS	4	W	
Colloca^34^	2008	14	22.3	100%	Electrical	Sham electrode	0-10 NRS	3	W	
Colloca^35^	2008a	16	32.0	66%	Laser	Inert gel	0-10 NRS	5	W	
Colloca^31^	2009	16	22.6	100%	Electrical	Sham electrode	0-10 NRS	5	W	
Colloca^36^	2019	107	28.1	64%	Electrical	Sham electrode	0-100 NRS	3	W	
De Pascalis^41^	2017	55	23.4	100%	Cold cup	Inert gel	0-100 NRS	3	W	
De Pascalis^42^	2019	58	24.5	100%	Cold cup	Inert gel	0-100 pain intensity scale	1	W	
Disley^45^	2021	50	21.0	87%	Cold pressor	Inert nasal spray	−5 to +5 VAS	1	W	
Ellingsen^51^	2013	28	25.5	33%	Thermal	Inert nasal spray	0-100 NRS	4	W	+
Fehse^54^	2015	27	32.0	0%	Thermal	Inert pill	0-10 pain intensity scale	5	B	
Geers^61^	2014	106	19.6	67%	Cold pressor	Inert gel	0-100 VAS	5	W	
Gniß^65^	2020	32	21.0	50%	Thermal	Inert gel	0-100 VAS	2	W	
Horing^73^	2020	17	19.6	54%	Thermal	Inert pill	0-10 VAS	2	W	
Hunter^75^	2014	15	27.0	100%	Electrical	Sham electrode	0-10 VAS	3	W	
Johnson^78^	1997	24	NR	50%	Cold pressor	Sham TENS	0-100 VAS	2	W	
Kube^84^	2020	25	23.6	44%	Thermal	Inert gel	0-100 VAS	2	W	
Locher^88^	2017	37	26.6	62%	Thermal	Inert gel	0-100 VAS	6	W	
Lyby^90^	2010	63	NR	48%	Thermal	Inert pill	0-100 VAS	2	W	
Lyby^91^	2011	33	22.0	51%	Thermal	Inert gel	0-10 NRS	1	W	
Lyby^92^	2012	33	22.0	30%	Thermal	Inert pill	0-10 NRS	2	W	
Matre^97^	2006	18	NR	41%	Thermal	Sham magnets	0-100 VAS	6	W	
Milling^101^	2009	41	NR	63%	Pressure	Inert gel	0-30 pain intensity scale	4	W	
Montgomery^103^	1996	56	NR	57%	Pressure	Inert gel	0-10 pain intensity scale	10	W	
Nemoto^107^	2007	10	NR	50%	Laser	Inert pill	0-10 pain intensity scale	3	W	
Nir^108^	2012	24	25.8	0%	Hot water	Inert pill	0-100 NRS	3	B	
Peerdeman^112^	2015	59	21.8	71%	Cold pressor	Inert pill	0-10 NRS	3	B	
Petrovic^115^	2002	9	NR	NR	Thermal	Inert pill	0-100 VAS	6	W	
Pontén^116^	2019	15	27	60%	Thermal	Sham electrode	0-100 NRS	2	W	
Rhudy^120^	2018	33	35.3	51%	Electrical	Inert gel	0-100 VAS	6	W	
Roelofs^121^	2000	30	21.6	0%	Electrical	Sham IV	0-100 VAS	5	B	
Rose^122^	2012	41	NR	61%	Cold pressor	Inert gel	0-10 VAS	6	B	
Skvortsova^133^	2018	54	22.1	100%	Cold pressor	Inert nasal spray	0-10 NRS	0	B	
Valentini^140^	2018	39	24.9	54%	Laser	Inert gel	0-10 NRS	2	B	
van Laarhoven^145^	2011	33	21.8	100%	Histamine	Inert gel	0-100 VAS	1	W	
Yeung^153^	2020	60	24.5	72%	Cold pressor	Inert gel	0-10 VAS	4	B	

For studies using a within-subject comparison to measure the placebo effect, N is reported as the number of participants from the group in which the comparison was made. For studies using a between-subject comparison, N is reported as the number of participants from the placebo and control groups from which the comparison was made or only the placebo condition in the case of within-subject comparisons.

C, control; IV, intravenous; N, sample size; NR, not reported; NRS, numeric rating scale; P, placebo; TENS, transcutaneous electric nerve stimulation; VAS, visual analogue scale.

**Table 3:** Characteristics of studies included in the observational learning (pain) meta-analysis.

Author	Year	N	Sample age	Percent female	Sensation induction method	Placebo manipulation	Rating scale	Risk of bias score	Comparison	Outlier based on funnel plot
Chen^22^*	2019	24	NR	52%	Thermal	Inert gel	0-100 VAS	3	W	
Chen^22^*	2019	43	NR	63%	Thermal	Inert gel	0-100 VAS	3	W	−
Chen^22^*	2019	30	NR	65%	Thermal	Inert gel	0-100 VAS	3	W	−
Colloca^31^	2009	16	22.6	100%	Electrical	Sham electrode	0-10 NRS	3	W	+
Hunter^75^	2014	30	27.0	100%	Electrical	Sham electrode	0-10 VAS	3	W	
Raghuraman^119^	2019	28	23.4	61%	Thermal	Inert gel	0-100 VAS	3	W	
Schenk^128^	2020	31	28.1	48%	Thermal	Inert gel	0-100 VAS	2	W	

For studies using a within-subject comparison to measure the placebo effect, N is reported as the number of participants from the group in which the comparison was made. For studies using a between-subject comparison, N is reported as the number of participants from the placebo and control groups from which the comparison was made, or only the placebo condition in the case of within-subject comparisons. In the final column, “−” indicates a potential outlier with an effect size smaller than the bounds of a 99% confidence interval, based on inspection of funnel plots (Supplementary Materials, http://links.lww.com/PAIN/B752).

* Three independent studies reported in a single article.

NRS, Numeric Rating Scale; VAS, visual analogue scale.

**Table 4:** Characteristics of studies included in the verbal suggestion (itch) meta-analysis.

Author	Year	N	Sample age	Percent female	Sensation induction method	Placebo manipulation	Rating scale	Risk of bias score	Comparison
Bartels^12^	2014	48	22.7	77%	Electrical	Sham electrode	0-10 VAS	4	B
Darragh^38^	2015	48	22	78%	Histamine	Inert gel	0-10 itch intensity scale	1	W
Peerdeman^112^	2015	59	21.8	71%	Histamine	Inert pill	0-10 NRS	3	B
Skvortsova^133^	2018	54	22.1	100%	Histamine	Inert nasal spray	0-10 NRS	0	B
van Laarhoven^145^	2011	36	21.8	100%	Histamine	Inert gel*	0-10 VAS	1	B
Meeuwis^99^	2019	45	23.2	83%	Histamine	Inert gel	0-10 NRS	4	W
Meeuwis^98^	2021	28	21.3	81%	Histamine	Sham patch	0-10 NRS	4	W

For studies using a within-subject comparison to measure the placebo effect, N is reported as the number of participants from the group in which the comparison was made. For studies using a between-subject comparison, N is reported as the number of participants from the placebo and control groups from which the comparison was made, or only the placebo condition in the case of within-subject comparisons.

* No actual inert gel was applied, instead the histamine gel was used and participants were led to believe an additional medical gel was added. No outliers based on inspection of funnel plots (Supplementary material, http://links.lww.com/PAIN/B752) were detected in this analysis.

N, sample size; NRS, numeric rating scale; VAS, visual analogue scale.

### 2.6. Statistical analyses

Analyses were conducted with the Comprehensive Meta-Analysis software, version 3.3.070 (Comprehensive Meta-Analysis, 2014) and R for visualizations (R Core Team, 2019). Given the heterogeneity of study designs and methods, a random-effects model was used for all meta-analyses. Effect sizes were calculated with means and SDs for each group (between-subject placebo vs control groups) or trial type (within-subject placebo vs control). If only difference scores with standard deviations for placebo vs control trials were reported, these were used instead. If only standard errors were reported, these were converted to standard deviations. For each included study, an effect size (Hedge *g*) weighted to the sample size (n) was computed*,* for which positive values indicate the presence of a placebo effect. Hedge *g* is a standardized parametric measure of effect size that represents the difference between 2 means in units of pooled standard deviations, commonly used in meta-analysis.^67,68^ It is similar to Cohen *d* but provides more accurate estimates of effect sizes for samples of less than n = 20, whereas the 2 perform equally well for samples of n > 20. Both can be interpreted on the same scale, in which values of approximately 0.20 can be considered small, 0.50 medium, and 0.80 large.^26^

#### 2.6.1. Primary outcome measure

The primary outcome measure was the magnitude of the placebo effect, defined as the difference in reported sensation intensity between placebo and control groups (between subjects) or trials (within subjects), when the intensity of the stimulus was equal across conditions (typically referred to as the test phase, evocation phase, or extinction phase in conditioning studies). The minimum group size for analysis was *k* = 3, where k denotes the number of included studies, based on a previous meta-analysis on a similar topic.^110^ Whenever possible, the mean of pain or itch ratings across the entire test phase was used because this was by far the most commonly reported outcome (Tables 1–4). If only values from the first trial(s) were reported, these were used instead. Sensitivity analyses tested for differences in placebo magnitudes between studies reporting the mean pain values for the entire evocation phase vs the first trials, where effects are thought to be strongest with less opportunity for extinction to occur. Similarly, for consistency, we used a within-subject comparison to measure the placebo effect when the necessary data were reported, and when this was not possible, between subjects or mixed within–between comparisons were used; we conducted sensitivity analyses to assess whether the type of comparison affected the placebo-effect magnitude. A Cochran *Q* test was used to measure the degree to which heterogeneity in placebo effect sizes could be explained by these factors.^25^ For within-subject comparisons, a prepost correlation value of 0.5 was used, and sensitivity analyses conducted by previous meta-analyses in related topics found that adjusting this value did not impact overall results.^110,139^ When a single study had more than one arm eligible for inclusion in a single meta-analysis (eg, a study comparing 2 classical conditioning paradigms with different numbers of trials,^33^ data from both arms were averaged across for the primary outcome but included separately for relevant subgroup analysis). Heterogeneity of resulting effect sizes was measured with *I*^*2*^, the proportional amount of variance in effect sizes attributed to heterogeneity between included studies,^70^ treating 0% to 40% as negligible, 30% to 60% as moderate, 60% to 90% as substantial, and 75% to 100% as considerable amounts of heterogeneity.^44^ Statistical significance of heterogeneity was measured with a Cochran *Q* test.

#### 2.6.2. Additional analyses

Subgroup analyses with random-effects models were used to explore differences between pain induction methods (eg, thermal pain, electrical pain) and placebo treatments (eg, placebo gels, electrodes). A Cochran *Q* test was used to measure the degree to which heterogeneity in placebo effect sizes could be explained by these subgrouping variables. Meta-regression was used to assess the impact of the number of acquisition trials, or instances in which a pain stimulus and associated placebo or control cue are paired during the acquisition phase of a classical conditioning paradigm. Similarly, meta-regression analysis was conducted for the number of trials in the evocation phase of a classical conditioning paradigm and for the difference in placebo and control stimulus intensity (during the acquisition phase of conditioning paradigms) when these were calibrated on the basis of subjective pain ratings. Meta-regression was also used to assess the potential impact of sample age and sex, measured in years and percentage female-identified participants, respectively. These demographic analyses were not preregistered and were conducted post hoc*.* All meta-regressions used mixed-effects models, and *Q* values are reported. For all additional analyses, the magnitude of the placebo effect served as the outcome variable. The minimum group size for subgroup analyses was *k* = 3.^110^

## 3. Results

### 3.1. Study selection

**Figure 1.**
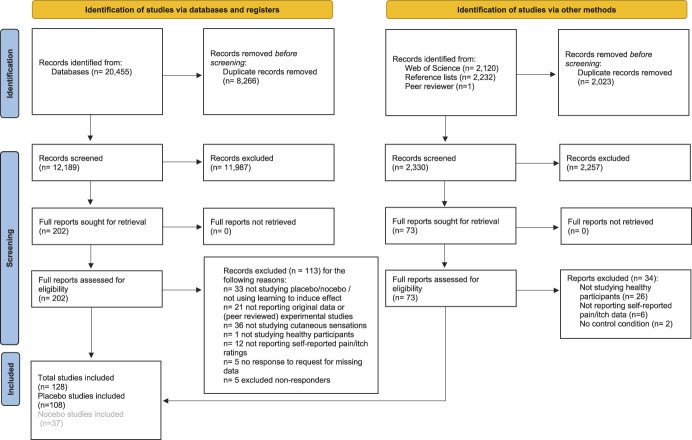
Flowchart of study inclusion process. Flow diagram of the inclusion and exclusion of studies for all searches. Nocebo studies are reported in a separate publication.

A total of 17,546 articles were identified through the initial database search (Fig. 1). After removal of 6672 duplicate results, 10,874 articles remained for consideration based on title and abstract. Of these, 174 articles remained whose full texts were reviewed, culminating in 80 articles initially included. The reference lists of these included articles were then screened (2232 referenced articles), yielding 17 more articles fit for inclusion. A Web of Science search for articles citing the 80 articles that were initially included then produced another 2120 articles for screening of which 22 articles fit all inclusion criteria. The database search was repeated in April 2020 and June 2021, ultimately yielding an additional 10 and 24 inclusions, respectively. During data extraction, 29 articles were excluded. In all, 24,814 articles were identified in various searches, 24,687 articles were excluded, resulting in 127 articles included; of which, 107 were included in placebo effect meta-analyses. One observational learning article was included during the revision process, bringing the total number of articles to 108. Details on inclusion and exclusions at each stage of the search can be found in Figure 1*.* Reasons for exclusions were assigned based on the first detected exclusion criteria to be violated.

### 3.2. Characteristics of included studies

Our search strategy identified 108 unique placebo studies, which met all inclusion criteria. Of these 108 studies, 68 had sufficient data for inclusion in the classical conditioning with verbal suggestions (CC + VS) on pain meta-analysis (Table 1), 39 had sufficient data for inclusion in the verbal suggestions alone (VS) on pain meta-analysis (Table 2), and 7 had sufficient data for inclusion in the observational learning on pain meta-analysis (Table 3). There are more arms than total unique studies because several studies that compared different learning processes had arms included in multiple meta-analyses. For itch, 7 studies had sufficient data for inclusion in the meta-analysis of verbal suggestions alone (Table 4). Only one study, Bartels et al.,^12^ was identified for classical conditioning with verbal suggestions on itch (the characteristics of this study are reported in Table 3 as a verbal suggestion arm of the study was included), and no studies for observational learning of placebo effects on itch, so no meta-analyses were conducted in these cases. The studies were published between the years 1996 and 2021. Several studies that met all inclusion criteria except the use of actual placebo intervention, and instead induced placebo effects with only abstract stimuli like color or shapes, were not included in the analysis (eg, Carlino et al.,^20^ Świder and Bąbel, ^132^ Bąbel et al.,^9^ Brączyk and Bąbel^16^). A record of inclusion and exclusion decisions can be found in the supplementary digital content for this article (available at http://links.lww.com/PAIN/B752), along with data extraction materials.

### 3.3. Primary outcome: magnitude of placebo effects

**Figure 2.**
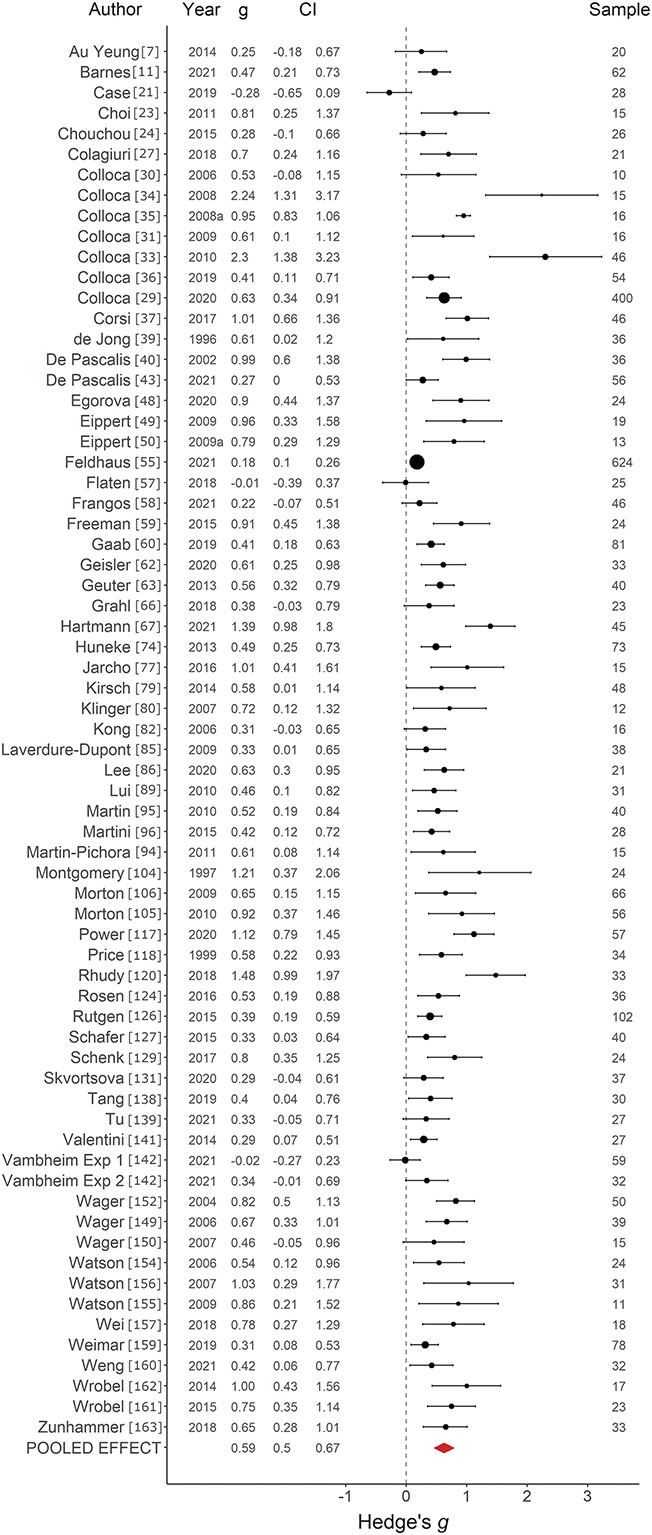
Forest plot of classical conditioning with verbal suggestion on pain studies. Forest plot depicting the magnitude of placebo effects on pain, measured with Hedge *g* in a random-effects model, in studies using a classical conditioning with verbal suggestion paradigm. CI, confidence interval.

**Figure 3.**
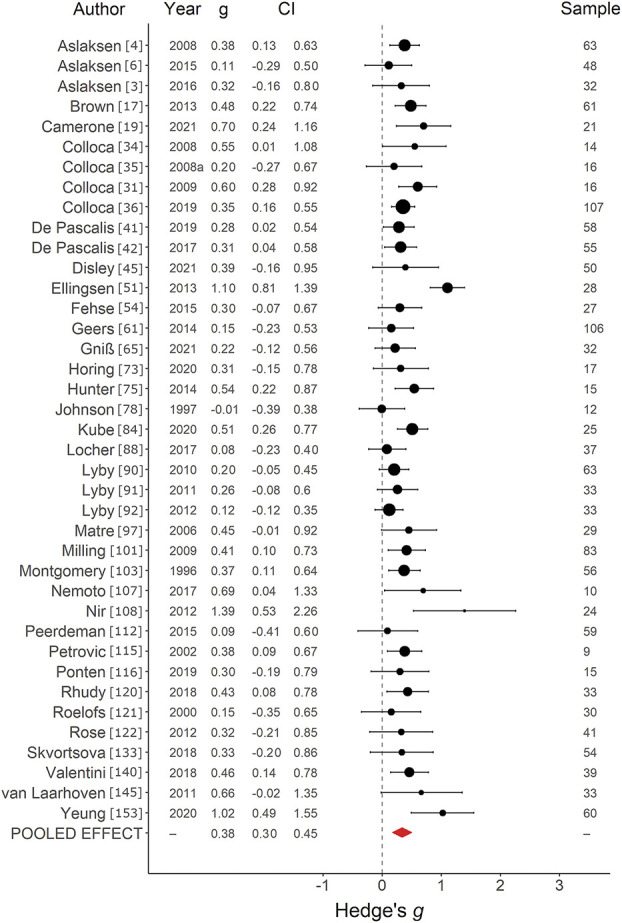
Forest plot of verbal suggestion on pain studies. Forest plot depicting the magnitude of placebo effects on pain, measured with Hedge *g* in a random-effects model, in studies using a verbal suggestion paradigm. CI, confidence interval.

**Figure 4.**
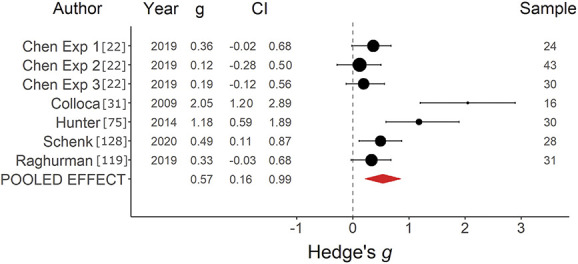
Forest plot of observational learning on pain studies. Forest plot depicting the magnitude of placebo effects on pain, measured with Hedge *g* in a random-effects model, in studies using an observational learning paradigm. CI, confidence interval.

**Figure 5.**
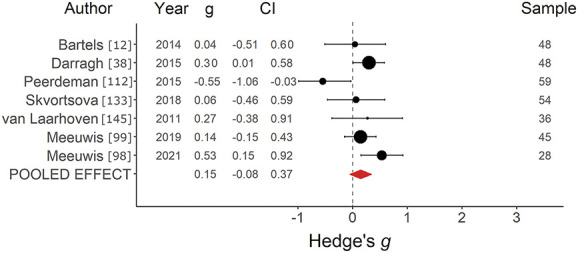
Forest plot of verbal suggestion on itch studies. Forest plot depicting the magnitude of placebo effects on itch, measured with Hedge *g* in a random-effects model, in studies using a verbal suggestion paradigm. CI, confidence interval.

Placebo effects on pain induced with CC + VS paradigms (*k =* 68) were found to have an average effect size of *g =* 0.59, SE *=* 0*.*04, 95% confidence interval (CI) *=* 0.50 to 0.67, *Q*(67) = 310.75, *P* < 0.001, *I*^2^ *=* 78.44%, indicating a medium positive effect with substantial heterogeneity (Fig. 2). Visual inspection of the funnel plot (Supplementary digital content Fig. S1, available at http://links.lww.com/PAIN/B752) indicated a likely effect of publication bias, and trim-and-fill method of Duval and Tweedie indicated that an estimated 22 studies were missing, resulting in an adjusted effect size of *g =* 0.41, 95% CI *=* 0.32 to 0.50. For placebo effects on pain induced with VS alone (*k =* 39), an average effect size of *g =* 0.38, SE *=* 0*.*04, 95% CI *=* 0.30 to 0.45, *Q*(38) *=* 65.64, *P =* 0.005, *I*^2^ *=* 40.58%, was found, indicating a small to medium positive effect with moderate heterogeneity (Fig. 3). Inspection of the funnel plot (Supplementary digital content Fig. S2, available at http://links.lww.com/PAIN/B752) indicated no clear risk of publication bias, and no studies were imputed with trim and fill. For placebo effects on pain induced with observational learning (*k =* 7), an average effect size of *g* = 0.57, SE *=* 0*.*21, 95% CI = 0.16 to 0.99, *Q*(6) *=* 26.72, *P <* 0.001, *I*^2^ *=* 77.50%, was found, indicating a medium positive effect, albeit from a relatively small sample of studies (Fig. 4), with substantial heterogeneity. Inspection of the funnel plot (Supplementary digital content Fig. S3), available at http://links.lww.com/PAIN/B752, indicated a potential risk of publication bias, and although no studies were imputed with trim and fill, this is inconclusive given the small sample size of studies. For placebo effects on itch induced with VS (*k =* 7), an average effect size of *g =* 0.14, SE *=* 0*.*12, 95% CI *=* −0.08 to 0.37, *Q*(6) *=* 12.16, *P =* 0.06, *I*^2^ *=* 50.78%, was found, indicating a small positive effect with moderate, marginally significant heterogeneity (Fig. 5). Inspection of the funnel plot (Supplementary digital content Fig. S4, available at http://links.lww.com/PAIN/B752) indicated no clear risks of publication bias, and no studies were imputed with trim and fill.

#### 3.3.1. Within-subject vs between-subject comparisons

Sensitivity analyses to assess whether our measurement of the placebo effect was impacted by the use of within-subject (*k =* 60, *g =* 0.59, 95% CI *=* 0.48-0.70) or between-subject (*k =* 8, *g =* 0.58, 95% CI *=* 0.41-0.75) comparisons revealed no effect in the CC + VS pain meta-analysis (*Q =* 0.02, *P =* 0.88). No differences for within-subject (*k =* 29, *g =* 0.38, 95% CI = 0.30-0.46) or between-subject (*k =* 10, *g =* 0.37, 95% CI *=* 0.16-0.58) comparisons were found in the VS pain meta-analysis (*Q =* 0.01, *P =* 0.92). It was possible to make a within-group comparison for all 7 studies in the observational learning pain meta-analysis. There was a substantial, marginally significant difference for within-subject (*k =* 3, *g =* 0.29, 95% CI *=* 0.10-0.48) and between-subject (*k =* 4, *g =* −0.07, 95% CI *=* −0.42 to 0.28) comparison in the VS itch meta-analysis (*Q =* 2.04, *P =* 0.08), although this should be interpreted with caution given the small sample size.

#### 3.3.2. Single or mean outcome measure

Sensitivity analyses assessed whether measuring a placebo effect on pain using the mean of all evocation phase trials or an initial subset of trials in CC + VS paradigms affected the magnitude of the effect. Most studies (*k* = 63, *g* = 0.60) reported using the mean of all evocation phase trials, whereas very few studies (*k =* 2, *g =* 0.46) reported using the first control and placebo trials, and *k =* 3 did not specify how the placebo effect was measured (Table 1).

### 3.4. Secondary outcomes: subgroup analyses

#### 3.4.1. Sensory induction method

From all included pain studies in the CC + VS meta-analysis, thermal stimulation was the most commonly used method of inducing pain (*k* = 35), with a medium effect size of *g =* 0*.*50 (95% CI = 0.38-0.62). This was followed by electrical stimulation with a medium-large effect size (*k =* 19, *g =* 0.77, 95% CI = 0.57-0.96) and laser stimulation with a medium effect size (*k =* 12, *g* = 0.61, 95% CI = 0.46-0.77). A *Q* test indicated that sensory induction method accounted for significant heterogeneity in the resulting placebo effect sizes (*Q*(2) = 7.2, *P =* 0.027), likely driven by differences between the thermal and electrical stimuli subgroups. For all included pain studies in the VS meta-analysis, thermal stimulation was again the most commonly used method of inducing pain with a medium-small effect size (*k =* 15, *g =* 0.34, 95% CI = 0.21-0.47), followed by cold pressor (*k =* 9, *g =* 0.33, 95% CI = 0.13-0.54), electrical stimulation (*k =* 7, *g =* 0.45, 95% CI = 0.32-0.57), and laser stimulation (*k =* 3, *g =* 0.42, 95% CI = 0.17-0.67), all small-to-medium effects. A *Q* test indicated that sensory induction method did not account for significant heterogeneity in the resulting placebo effect sizes (*Q*(3) = 3.78, *P =* 0.28). For studies in the observational learning meta-analysis, thermal stimulation was the most commonly used method for inducing pain with a small effect size (*k* = 5, *g =* 0.25, 95% CI = 0.12-0.39) and the only subgroup to meet the *k* = 3 threshold. In the itch VS meta-analysis, itch was most often induced with histamine (*k =* 6, *g =* 0.15, 95% CI = −0.10 to 0.41), yielding a small effect size, whereas one study used electrical stimulation. With only 1 subgroup meeting the *k* = 3 threshold, no *Q* test was conducted.

#### 3.4.2. Placebo treatment

From all included pain studies in the CC + VS meta-analysis, inert gels, creams, and lotions applied on the skin where the pain stimuli would later be administered were the most common form of placebo treatment (*k =* 44). Studies using this form of sham treatment had an average placebo effect size of *g =* 0.40, 95% CI *=* 0.36 to 0.45. This treatment was followed by sham electrodes and transcutaneous electric nerve stimulation (TENS) devices (*k =* 18), which yielded a larger average placebo effect across studies (*g =* 0.67, 95% CI = 0.59-0.74). Other placebo treatments (pill, injection, nasal spray, acupuncture) did not meet the k ≥ 3 group size threshold. A *Q* test indicated that the type of placebo intervention accounted for significant heterogeneity in the resulting placebo effect sizes (*Q*(2) = 14.05, *P =* 0.007). From pain studies included in the VS-alone meta-analysis, inert gel was again the most common treatment (*k =* 17) with an average placebo effect size of *g =* 0.34 (95% CI *=* 0.26 to 0.41). Placebo pills were the next most commonly used treatment (*k =* 9, *g =* 0.29, 95% CI *=* 0.17-0.41). Sham electrodes and TENS devices (*k =* 6) again yielded a larger effect than inert gels (*g =* 0.47, 95% CI *=* 0.32-0.63) but with overlapping confidence intervals. A *Q* test indicated that the type of placebo intervention did not account for significant heterogeneity in the resulting placebo effect sizes (*Q*(3) = 1.35, *P =* 0.51). From pain studies included in the observational learning meta-analysis, inert gel was the most common treatment (*k* = 5) with an average placebo effect size of *g =* 0.25 (95% CI = 0.12 to 0.39), and the only treatment to meet the *k* = 3 threshold. From itch studies included in the VS-alone meta-analysis, inert gel was the most common treatment (*k* = 3) with a small effect size of *g =* 0.22 (95% CI = 0.03 to 0.42) and the only treatment to meet the *k* = 3 threshold.

#### 3.4.3. Length of acquisition and evocation phases

From the 68 arms included in the pain CC + VS meta-analysis, 65 reported how many pain stimulus trials were used in the acquisition phase. The mean number of pain stimulus trials used in an acquisition phase for a CC + VS paradigm was 26 (SD *=* 18.70) and ranged from 2 to 112 placebo and control trials summed. Meta-regression indicated that the length of the acquisition phase did not explain the heterogeneity in resulting placebo effect sizes (*Q =* 1.94, *P =* 0.16). Similarly, 66 included studies reported how many pain stimulus trials were used in the evocation phase. The mean number of pain stimulus trials used in an evocation phase for a CC + VS paradigm was 21 (SD *=* 15.40) and ranged from 2 to 60 trials (placebo and control trials summed). In studies that calculated the placebo effect with the mean pain values from the entire evocation phase (*k =* 62), the length of the evocation phase did not explain the heterogeneity in resulting placebo effect sizes (*Q =* 1.89, *P =* 0.17).

#### 3.4.4. Difference in placebo and control stimulus intensity during acquisition

From the 68 arms included in the pain CC + VS meta-analysis, 49 used individually calibrated pain intensities for placebo and control stimuli during the acquisition phase. On a 0 to 100 pain intensity scale, the mean calibrated difference in placebo and control stimuli was 36 (SD *=* 8.8) with differences ranging from 20 to 60 points. The difference in calibrated pain intensity for placebo and control acquisition trials did not explain the heterogeneity in resulting placebo effect sizes (*Q =* 0.70, *P =* 0.40).

#### 3.4.5. Sex of participant samples

From the 68 arms included in the pain CC + VS meta-analysis, 67 reported the sex distribution of their sample. Sex distributions ranged from 0% to 100% female, with a mean of 54% female identified (SD = 25.4). Participant sex was not found to explain the heterogeneity in placebo effect sizes for the pain CC + VS studies (*Q* = 0.19, *P* = 0.66). From the 39 arms included in the pain VS-alone meta-analysis, 37 reported the sex distribution of their sample. Sex distributions ranged from 0% to 100% females with a mean of 59.5% females identified (SD = 27.0). Participant sex was not found to explain the heterogeneity in placebo effect sizes for the pain VS-alone studies (*Q* = 0.00, *P* = 0.95). From the 7 arms included in the observational learning pain meta-analysis, each study reported sex distribution of the sample. Sex distribution ranged from 48% to 100% females with a mean of 69.8% females identified (SD = 21.4) Meta regression indicated that sex distribution of the sample could explain heterogeneity in placebo effect sizes, with a higher percentage of female participants corresponding to larger placebo effects (*Q* = 8.34, *P* = 0.004). This finding should be interpreted with caution given the small sample of studies it was derived from. From the 7 arms included in the itch VS-alone meta-analysis, each study reported sex distribution of the sample. Sex distribution ranged from 71% to 100% females with a mean of 84.3% females identified (SD = 11.4). Participant sex was not found to explain the heterogeneity in placebo effect sizes for the observational learning pain studies (*Q* = 0.50, *P* = 0.48).

#### 3.4.6. Age of participant samples

From the 68 arms included in the pain CC + VS meta-analysis, 58 reported the mean age. Mean age ranged from 19.3 to 49.0, with a mean across arms of 25.4 years (SD = 5.0). Participant age was not found to explain the heterogeneity in placebo effect sizes for the pain CC + VS studies (*Q* = 1.14, *P* = 0.28). From the 39 arms included in the pain VS-alone meta-analysis, 30 reported the mean age. Mean age ranged from 19.5 to 32.0 years with a mean age across arms of 23.8 years (SD = 3.2). Participant age was not found to explain the heterogeneity in placebo effect sizes for the pain VS-alone studies (*Q* = 0.14, *P* = 0.70). From the 7 arms included in the observational learning pain meta-analysis, 4 studies reported mean age of the sample. Mean age ranged from 22.6 to 28.5 with a mean across arms of 25.3 years (SD = 2.7). Participant age was not found to explain heterogeneity in placebo effect sizes for pain observational studies (*Q* = 0.37, *P* = 0.55). From the 7 arms included in the itch VS-alone meta-analysis, each study reported the mean age of the sample. Mean age ranged from 21.3 to 23.2 years with a mean across arms of 22.1 years (SD = 0.6). Participant age was not found to explain heterogeneity in placebo effect sizes for itch VS-alone studies (*Q =* 0.20, *P =* 0.65).

#### 3.4.7. Risk of bias

Across included studies, risk of methodological bias measured with the Marcuzzi risk of bias tool for quantitative sensory testing^91^ was found to be low overall (Tables 1–4). In the pain CC + VS meta-analysis, a relationship between risk of bias cores and placebo effect size was detected (*Q =* 4.37, *P =* 0.04), such that higher risk of bias scores corresponded with larger placebo effect sizes. In the VS-alone pain meta-analysis, no relation between risk of bias scores and placebo effects was detected (*Q =* 0.19, *P =* 0.66). Similarly, no relationship was detected for observational learning pain studies (*Q =* 0.22, *P =* 0.64) or for VS-alone itch studies (*Q =* 0.00, *P =* 0.98).

## 4. Discussion

Our systematic review and meta-analysis of experimentally induced placebo effects on cutaneous pain and itch in healthy participants primarily investigated how different learning mechanisms (classical conditioning, verbal suggestion, observational learning) contributed to placebo effect magnitudes. Additionally, we explored whether sensation induction method (thermal pain, electrical pain, etc), type of placebo treatment (inert gel, electrodes, etc), number of acquisition or evocation trials in a classical conditioning paradigm, calibrated intensity between placebo and control stimuli, and sex and age of the sample can impact the magnitude of placebo effects on cutaneous pain and itch. With 108 included studies from 1996 to 2021, this review offers a comprehensive and systematic assessment of 25 years of experimental placebo research in healthy human participants.

The primary meta-analyses indicated that conditioning with verbal suggestion induced placebo effects on pain (*k =* 68, *g =* 0.59) of a medium effect size. This is larger than for verbal suggestion alone (*k =* 39, *g =* 0.38), underscoring the premise that when attempting to harness positive treatment expectations to yield better treatment outcomes, diverse and recurrent learning processes will be most effective. However, this estimate is notably smaller than the large pooled effect size found in a previous meta-analysis of experimental placebo analgesia studies published between 2002 and 2007 (*d =* 1.00), in which effects from studies using different learning processes and patient samples were analyzed together. It appears that the studies included in the current meta-analysis reported smaller placebo effects on average. This may be a product of better scientific practices like preregistration or open science reducing the preponderance of biased results or better experimental design yielding more accurate results. Then again, the trim-and-fill results suggested a bias in these findings, with a likelihood of missing or unreported studies with underwhelming results. Taken with the positive correlation between risk of bias scores and effect sizes in this meta-analysis, the potential for biased methodology, analysis, and reporting, at least in some studies, cannot be ruled out. However, risk of bias scores were generally low and with a sample of 68 studies from numerous laboratories, several biased results or unpublished null findings are not likely to have a major impact on the overall placebo effect size.

Our meta-analysis of verbal suggestion alone studies yielded a smaller placebo effect size on pain (*k =* 39, *g =* 0.38) than for verbal suggestion with conditioning, replicating previous findings from several individual experiments and a related meta-analysis of nocebo effects.^28,34,36,111^ Verbal suggestion studies typically measure placebo effects across a much smaller number of stimuli than conditioning studies, making a comparison between the 2 methods difficult when taking into account the potential for extinction over numerous stimuli. It is likely that besides being smaller than a placebo effect induced with a classical conditioning paradigm, effects induced with verbal suggestion alone may also be more rapidly extinguished without reinforcement. Although we found that placebo effects on pain for studies using observational learning (*k =* 7, *g =* 0.57) were comparable in magnitude to those induced with classical conditioning combined with verbal suggestion, the somewhat small sample size of included studies and lack of specific search terms for observational learning, indicating possible missing studies, may skew the results.

The pooled effect size for placebo effects on itch induced with verbal suggestion (*k =* 7, *g =* 0.14) was notably smaller than the pooled effect size for placebo effects on pain induced with verbal suggestion (*k =* 39, *g =* 0.38), although with overlapping confidence intervals. Although there were not enough studies to make meaningful comparisons between learning methods for itch across studies, within one study that compared classical conditioning with verbal suggestion to verbal suggestion alone for placebo effects on itch found the same pattern of results as seen in pain.^12^ In contrast to a study in which placebo effects on pain and itch were directly compared,^140^ verbal suggestion for placebo effects on itch appears to yield smaller effects relative to those on pain. Itch induction methods are generally less precise than those for pain,^15^ which may make it more challenging for itch studies to reliably induce sensations at a consistent intensity, but it is not clear if that explains the smaller effect size. The underlying biological mechanisms of these effects may also contribute to this difference. Although placebo effects on pain appear to be substantiated in part by endogenous opioids,^129^ demonstrated by the blockade of placebo effects by opioid antagonists,^1,14^ itch appears to increase following administration of opioid agonists.^75^ If placebo effects for other sensations like itch also cooccur with endogenous opioid release, this could explain their smaller effect size relative to placebo effects on pain, as the opioid release blunts the antipruritic placebo effect. However, it remains an open question whether placebo effects on itch are substantiated by endogenous opioids in the same way that placebo effects on pain are. Pathways specific to the itch induction method (eg, histaminergic) could instead play the role that opioid pathways play for pain.

For sensation induction methods in pain, across both classical conditioning with verbal suggestion and verbal suggestion–alone analyses, studies using laser and electrical pain induced larger placebo effects than the more commonly used thermal pain; however, the 95% confidence intervals for these effect size estimates do overlap. Laser and electrical pain stimuli are both generally much shorter in duration than thermal stimuli, with durations typically less than 300-millisecond long^103,117^ and thermal stimuli typically 5- to 15-second long.^48,55^ It is possible that the intensity of such short stimuli are harder for the participant to assess, allowing more room for the expectancies thought to underlie placebo effects to shape the pain experience. According to predictive coding models, these short and less precisely experienced stimuli could give way to a larger effect of prior expectations on the sensory experience.^18^ Alternatively, thermal pain may be more familiar to participants, and familiar sensations could be easier to assess and less influenced by expectancies.^61^ Cold pressor, another long duration and potentially familiar pain stimulus, typically endured for 1 to 3 minutes^109,128^ also yielded smaller placebo effects than laser and electrical pain, in line with this supposition.

Regarding the type of placebo treatment, studies that made use of sham electrodes induced larger placebo effects than those using topical gels or creams in the classical conditioning with verbal suggestion pain meta-analysis. A similar pattern was observed in the VS-alone pain meta-analysis, albeit with overlapping 95% confidence intervals. Speculatively, electrodes may have been seen as a more convincing treatment for pain than gels and ointments in the eyes of participants, contributing to greater perceived pain reductions. One study that investigated expectations for pain relief from various forms of treatment found that topical treatments like gels and creams were viewed as less effective than oral or injected treatments, but electrical stimulation was not included as a treatment in this survey.^108^ Some studies (eg, Colagiuri and Quinn,^27^ Barnes et al.^11^) allowed participants to feel several electrical or tactile stimuli through the electrodes before surreptitiously deactivating them, which may enhance the believability of the placebo treatment.

The other planned subgroup analyses on methodological factors did not explain differences in the magnitudes of placebo effects between the studies. We expected the number of trials used in either the acquisition or evocation phases of a classical conditioning paradigm to play a role in shaping the magnitude of placebo effects. More trials during acquisition could be expected to produce a stronger effect and one that is more robust to extinction.^33,124^ More trials during evocation, inversely, could dilute the effect when taking a mean across all trials because this would allow more time for extinction to take place. Instead, we found no evidence that either metric was related to placebo magnitudes. However, studies that had longer acquisition phases often had longer evocation phases as well, so the two may have cancelled each other out. If future studies were to report or make available data at a trial-by-trial level instead of only the mean of the acquisition or evocation phase, a more nuanced understanding of acquisition and extinction process for placebo effects could be realized. Although a larger calibrated difference in placebo and control stimulus intensities during acquisition could be expected to induce a larger placebo effect at evocation,^63^ our results did not support this notion. In a predictive coding framework, larger differences between placebo and control stimuli during acquisition should induce expectations for greater reductions in pain, allowing for larger placebo effects, within a certain limit.^18^ Too large of a difference between the expected and actual intensity of a pain stimulus could diminish the effect of expectation on pain perception.^71,107^ However, we saw no evidence for this in our results.

Regarding our post hoc investigation of possible moderating effects of sample sex and age, we did not observe any significant relationships between either demographic variable and placebo effects on pain or itch. Two recent systematic reviews of sex differences in experimental placebo and nocebo studies that compared placebo responses by females with those of males as part of their planned analyses found evidence that men show stronger placebo effects on pain than women, particularly when only verbal suggestions are used.^52,138^ However, this effect was not replicated by our meta-regression, which was not restricted to studies that reported on tests of sex differences in placebo effects. Although sex differences have not been explored for placebo effects on itch, one study to investigate differences for nocebo effects on itch found no effect of sex.^130^ The relationship between participant age and placebo effects on pain or itch has not been explored across the adult lifespan. Some recent studies have compared placebo effects between child and adult samples^152,155^ but not between different stages of adulthood. The samples included in these meta-analyses skewed strongly toward young adults in their late teens to mid-20s, limiting an exploration of placebo effects in older adult and geriatric populations. Given the elderly's increased utilization of health care, a better understanding of placebo effects and treatment expectancies in this population could be especially useful, yielding clinically relevant outcomes like improved treatment efficacy through psychological interventions on top of routine care.

Finally, risk of bias measured with the Marcuzzi risk of bias tool was checked as a possible predictor of placebo magnitudes. Generally, studies in this review gave more than sufficient detail on their samples, methods, and results, with low risk of bias overall. Although there was a relationship between larger effect sizes reported in studies with a greater risk of bias in the CC + VS pain meta-analysis, there was a skew toward nearly all studies having a low risk of bias. Furthermore, it should be noted that the Marcuzzi risk of bias tool, whereas specifically designed to assess risk of bias in quantitative sensory testing research, did not measure potential sources of bias such as random allocation of participants into conditions, and it may be useful to augment the tool with the Cochrane risk of bias tool in future work.^69^

Although this review advances our grasp of the mechanisms underlying placebo effects, considerable heterogeneity in the results remains unexplained. Other variables that may explain some of the heterogeneity, but were not investigated in the current meta-analysis, include demographic characteristics, the inclusion and exclusion criteria used to screen participants, individual differences, and methods unique to individual laboratory results. Regarding demographic characteristics, samples may vary in what proportion of participants are university students, particularly psychology or medical students, and such participants may be more skeptical of placebo manipulations than nonuniversity populations. Considering inclusion and exclusion criteria, and methods specific to individual laboratory results, these potential effects are harder to ascertain because they are not always clearly reported. For example, listing current physical or mental illness as an exclusion criterion is common practice in placebo research, but how these illnesses are screened for (eg, self-report, diagnostic interview or tests) may vary considerably between laboratory results and experiments. Similarly, other laboratory and experimenter effects (eg, demeanor of the experimenter, if the laboratory is set in a hospital vs an academic building, setup of the laboratory, etc.) are often not clearly documented or reported and may contribute to some of the heterogeneity in the placebo effect magnitudes reported here.

Future research, both systematic and at the level of individual studies, would do well to measure the variables described in the previous section, when possible. Similarly, future experimental work on placebo effects for cutaneous sensations could strengthen the field by attempting to replicate and expand on the few findings related to how variables such as gender, number of acquisition or evocation trials in classical conditioning paradigms, and calibrated difference between placebo and control stimuli can moderate placebo effects. In addition, methodological factors that were not investigated in this review, such as the intensity of pain or itch stimuli during the evocation phase, and differences between other pain modalities (eg, ischemic, visceral) could also be explored systematically and in individual experiments to build a more complete understanding of the factors that shape placebo effects. Much heterogeneity in placebo effect magnitudes remains unexplained, and investigating nonlinear interactions between experimental manipulations and individual differences may yield new insights, as indicated by the work of Hird et al.^71^ on boundary effects. Greater adoption of open science practices like the sharing of datasets, protocols, and other materials would also strengthen the field as a whole, allowing, amongst others, for more in-depth meta-analyses and reviews.

To conclude, in this systematic review and meta-analysis, we demonstrate the robust occurrence of substantial placebo effects on cutaneous pain and itch following various learning processes. We replicated previous findings showing that the size of these effects depends on the learning process used to induce them, with a combination of classical conditioning with verbal suggestion for placebo effects on pain induced a medium-sized effect, larger than the small effect seen from verbal suggestion alone. Verbal suggestions for placebo effects on itch appeared to induce smaller effects than those seen in pain, and more research is needed to understand this difference. Although differences in methodology and sample demographics generally did not seem to impact the placebo effect, consideration related to the believability of the experimental procedure, such as the type of sensation induction and placebo treatment used, may boost the efficacy of learning mechanisms used to induce these effects. The lack of influence from other methodological and demographic factors highlights how robust placebo effects are, suggesting that the learning mechanisms underlying these effects may be applied in settings outside the laboratoery to enhance our sensory experience.

## Conflict of interest statement

The authors have no conflict of interest to declare.

## Appendix A. Supplemental digital content

Supplemental digital content associated with this article can be found online at http://links.lww.com/PAIN/B752.
