# Supplementary material for: Placebo effects on cutaneous pain and itch: a systematic review and meta-analysis of experimental results and methodology
Source: Pain. 2022 Nov 16;164(6):1181–99. doi: 10.1097/j.pain.0000000000002820 (PMC10184563; doi:10.1097/j.pain.0000000000002820)
Supplement: Supplementary file 1 [file jop-164-1181-s001.pdf]

Supplementary Digital Content

Search strategy by database

PubMed:

(“Placebo Effect”[Mesh] OR "Placebo effect"[Title/Abstract] OR "Placebo effects"[Title/Abstract] OR "Placebo response"[Title/Abstract] OR "Placebo responses"[Title/Abstract] OR "Placebo analgesia"[Title/Abstract] OR "Placebo analgesic"[Title/Abstract] OR "Nocebo Effect"[Mesh] OR Nocebo[Title/Abstract] OR Expectancy[Title/Abstract] OR Expectancy[Title/Abstract] OR "Conditioning (Psychology)"[Mesh:noexp] OR "Conditioning, Classical"[Mesh] OR "Conditioning, Operant"[Mesh] OR (conditioning[tiab] NOT medline[sb]) OR “Verbal Suggestion”[Title/Abstract] OR “Verbal Suggestions”[Title/Abstract]**) AND (**"Pain"[Mesh] OR "Analgesia"[Mesh] OR Pain[Title/Abstract] OR Nocicept*[Title/Abstract] OR Analges*[Title/Abstract] OR Hyperalges*[Title/Abstract] OR Hypoalges*[Title/Abstract] **OR** “pruritus”[Mesh:noexp] OR “antipruritics”[Mesh] OR Itch[Title/Abstract] OR Pruritus[Title/Abstract] OR Pruritic[Title/Abstract]**) NOT (**"animals"[Mesh] NOT "humans"[Mesh]**)**

PsycINFO

(placebo/ OR conditioning/ OR classical conditioning/ OR (placebo effect OR placebo effects OR placebo response OR placebo responses OR placebo analgesia OR Nocebo OR Expectancy OR Expectancies OR conditioning OR verbal suggestion OR verbal suggestions).ti,ab.**) AND (**pain/ OR analgesia/ OR (pain OR analgesia OR analgesic OR hyperalgesia OR hyperalgesic OR hypoalgesia OR hypoalgesic OR itch OR pruritus OR pruritic).ti,ab.**)**

EMBASE

((placebo effect OR placebo effects OR placebo analgesia OR placebo response OR placebo responses OR nocebo OR Expectancies OR conditioning OR verbal suggestion OR verbal suggestions) **AND** (pain OR analgesia OR analgesic OR hyperalgesia OR hyperalgesic OR hypoalgesia OR hypoalgesic OR itch OR pruritus OR pruritic)**).**ti,ab. **NOT (**(nonhuman/ OR animal/) NOT human/**)**

Cochrane CENTRAL Methodology Register

([mh "Placebo Effect"] OR [mh ^"Conditioning (Psychology)"] OR [mh "Conditioning, Classical"] OR ("placebo effect" OR "placebo effects" OR "placebo analgesia" OR Nocebo OR expectancy OR expectancies OR conditioning OR "verbal suggestion" OR "verbal suggestions":ti,ab,kw)**) AND (**[mh Pain] OR [mh Analgesia] OR (pain OR analgesia OR analgesic OR hyperalgesia OR hyperalgesic OR hypoalgesia OR hypoalgesic OR itch OR pruritus OR pruritic:ti,ab,kw)**)** NOT **(**[mh animals] NOT [mh humans]**)**

Example scoring of Risk of Bias form based on Marcuzzi et al., 2013

LOW 5/34

Appendix B – Risk of bias assessment tool

**CRITERIA**	**YES**	**NO**	**UNCLEAR**	**NA***	
Inclusion/exclusion criteria comprise clear information about:					
Duration of pain (stage i.e. acute, subacute)	✓				No points
Age of participants included (cut-off)	✓				
Diagnosis description				✓	No points
**Final decision: Were inclusion/exclusion criteria clearly specified?**<br>Comment:	✓				
Clinical/demographical characteristics include full information about:					
Sample size	✓				
Mean age of participants		✓			2 points
Gender percentages	✓				
Mean pain duration at time of assessment(s)	✓				
Mean pain intensity at time of assessment(s)	✓				
**Final decision: Were clinical/demographical variables fully described?**<br>Comment:	✓				
The recruitment procedure include information about:					
Clinical setting(s)				✓	
How patients have been recruited (e.g. consecutive, convenience sample)	✓				
Controls/comparators recruitment				✓	
**Final decision: Was recruitment procedure specified and appropriate?**<br>Comment:	✓				
Somatosensory assessment:					
**Was method used to assess somatosensory function standardized or validated?**<br>Comment:	✓				
Method of somatosensory assessment include information about:					
Number of examiner involved in the assessment (where appropriate)		✓			
Equipment/tool used	✓				
Comprehensive description of the procedure (where appropriate)	✓				
Number of assessments	✓				
Assessment sites	✓				
**Final decision: Was somatosensory assessment fully described?**<br>Comment:	✓				
Blinding of assessment:					
**Were examiners who carried out somatosensory assessment blinded to participant group or condition?**<br>Comment:			✓		Not noted 1 point
Controlled risk of known confounders:					
Confounders know to influence somatosensory assessment were evaluated	✓				
A description of how confounders have been handled was provided	✓				
**Final decision: Were factors known to influence somatosensory assessment evaluated or controlled for?**<br>Comment:	✓				

*NA: not applicable

Figure S1

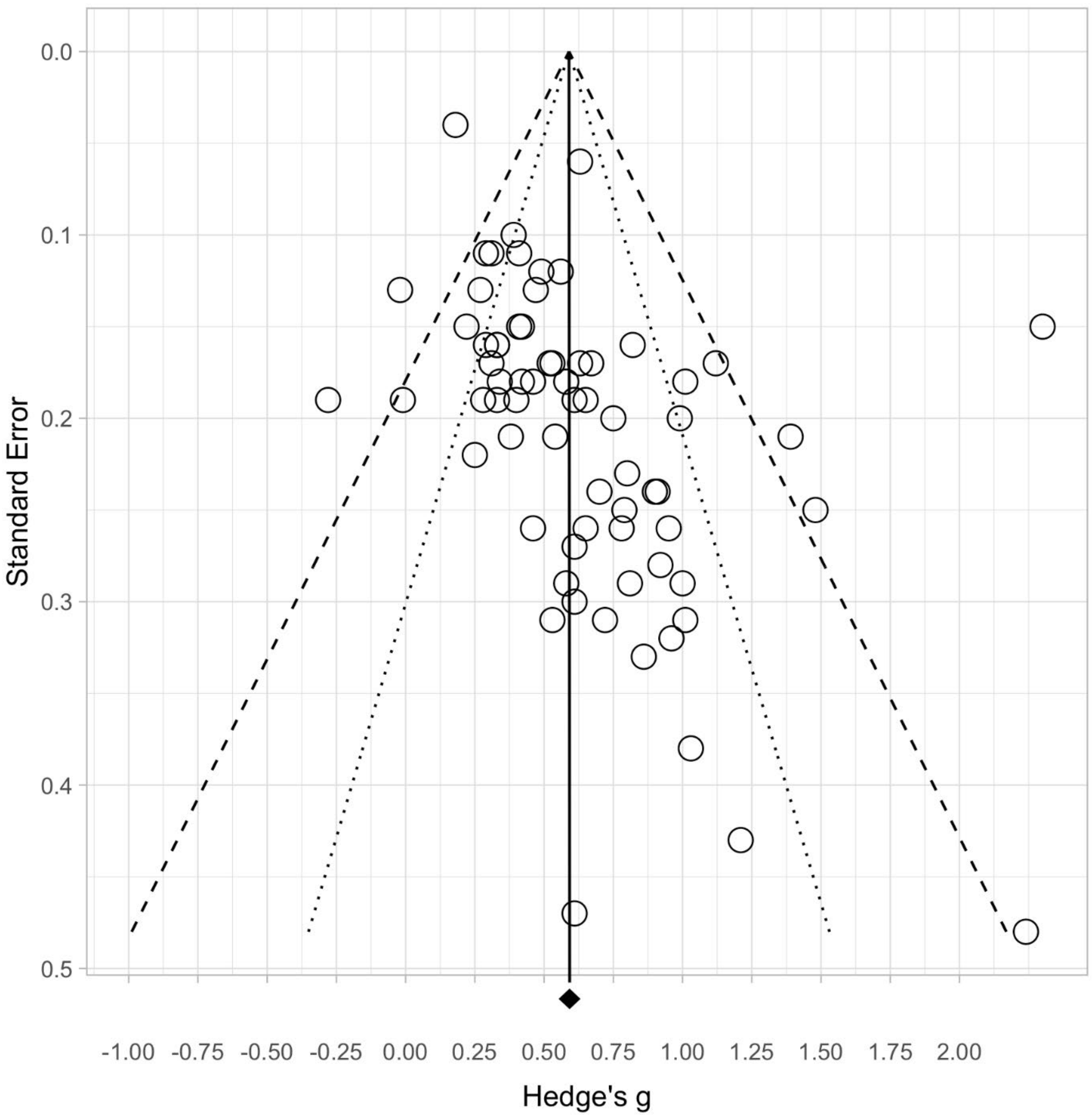

Note. Funnel plot of studies included in the pain classical conditioning with verbal suggestion meta-analysis. Outer, dashed line indicates a 95% confidence interval. Inner, dotted line indicates a 99% confidence interval.

Figure S2

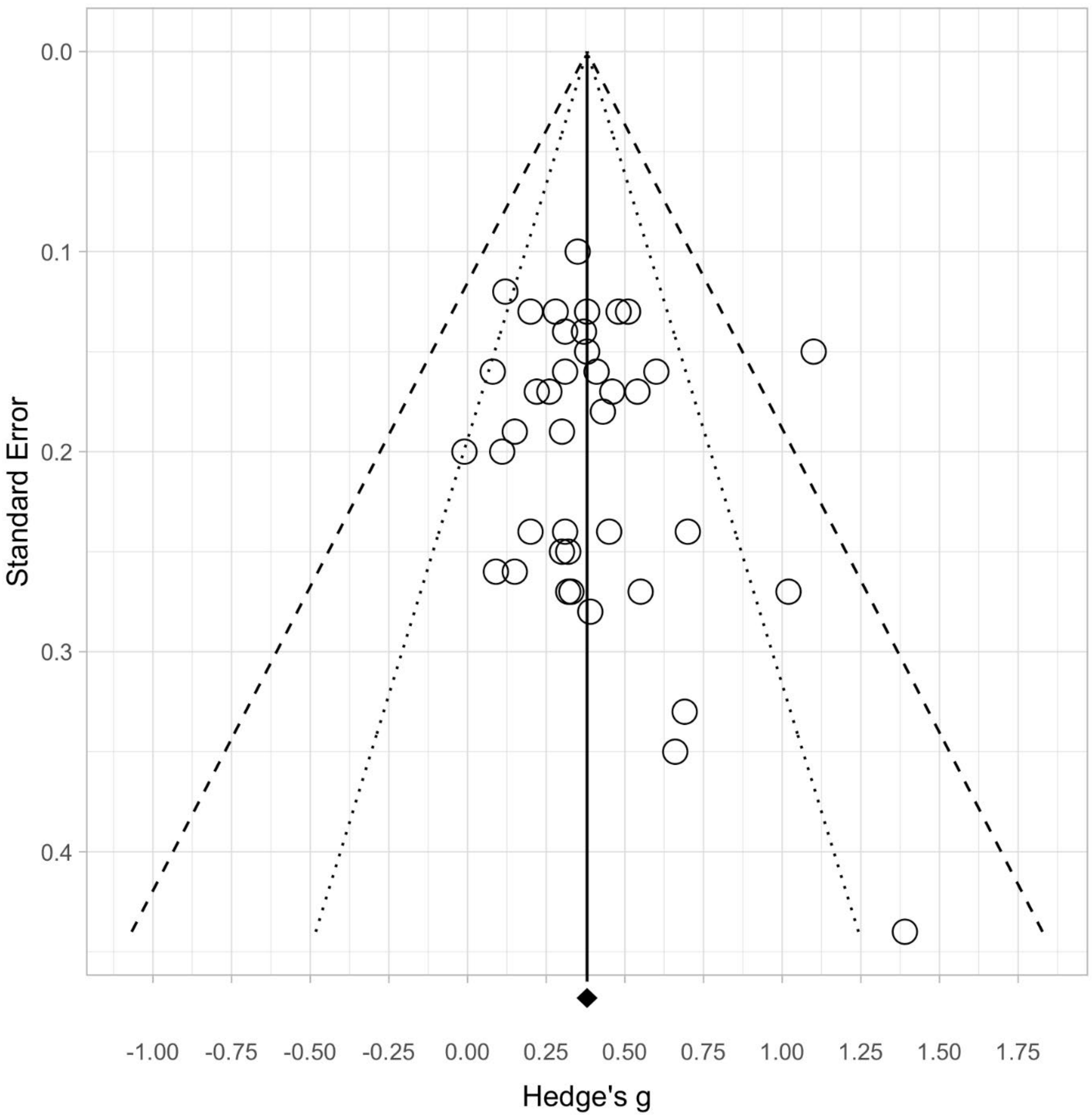

Note. Funnel plot of studies included in the pain verbal suggestion meta-analysis. Outer, dashed line indicates a 95% confidence interval. Inner, dotted line indicates a 99% confidence interval.

Figure S3

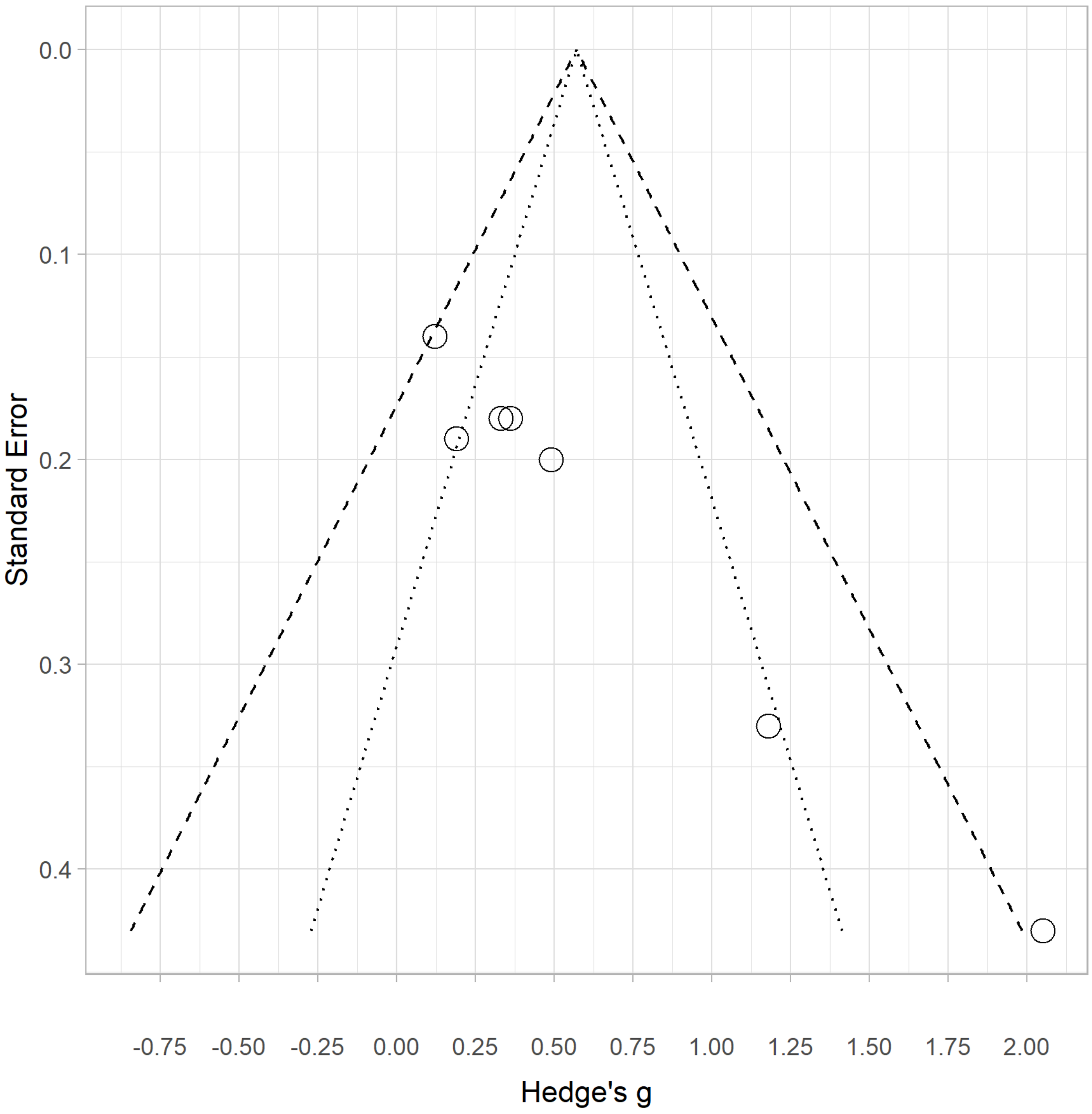

Note. Funnel plot of studies included in the pain observational learning meta-analysis. Outer, dashed line indicates a 95% confidence interval. Inner, dotted line indicates a 99% confidence interval.

Figure S4

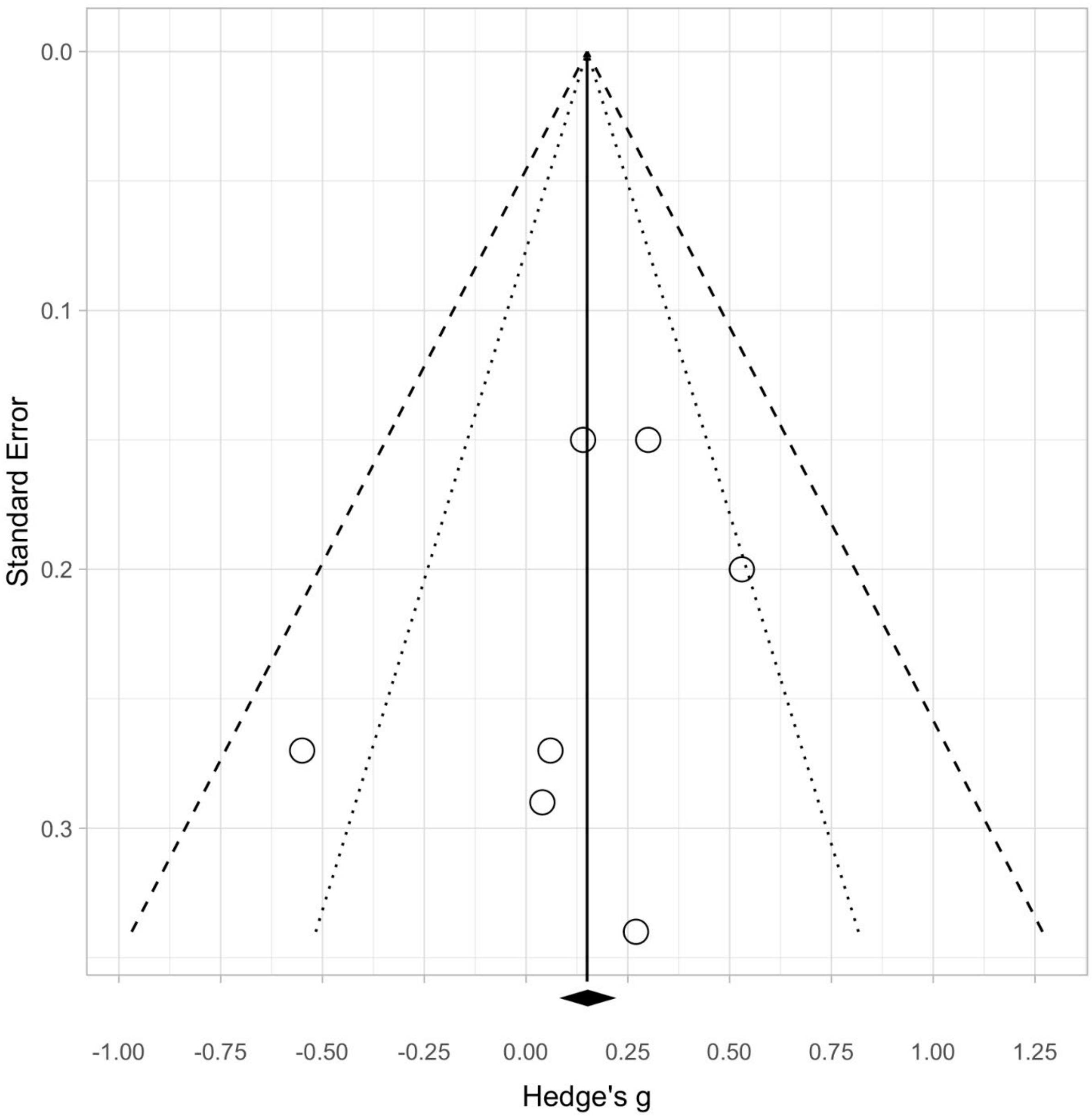

Note. Funnel plot of studies included in the itch verbal suggestion meta-analysis. Outer, dashed line indicates a 95% confidence interval. Inner, dotted line indicates a 99% confidence interval.

Author	Year	Title	Total N	M/F ratio	Mean age (years)	SD age (years)	Learning method (active condition)	Symptom induction method	Control condition	Comparison (Within, between, W-B)
Aslaksen, P. M., Zwarg, M. L., Eilertsen, H. I. H., Gorecka, M. M., Bjørkedal, E. and Bjørkedal, E.	2015	Opposite effects of the same drug: reversal of topical analgesia by nocebo	142	69M / 73F	23.4	4.1	VS	Thermal pain	Natural history (no VS)	B
Aslaksen, P. M., Åsli, O., Øvervoll, M. and Bjørkedal, E.	2016	Nocebo hyperalgesia and the startle response	61	28M / 33F	21.64	3.3	VS	Thermal pain	Natural history (no VS)	B
Aslaksen PM, Flaten MA.	2008	The roles of physiological and subjective stress in the effectiveness of a placebo on experimentally	63	31M / 32F			VS	Thermal pain	Natural history (no VS)	W-W
Bąbel, Przemysław, Adamczyk, Wacław, Świder, Karolina, Bajcar, Elżbieta A., Kicman, Paweł and Lisińska, Natalia	2018	How classical conditioning shapes placebo analgesia: Hidden versus open conditioning	90	0M / 90F	23.51	2.72	CC	Electrical pain	Sham conditioning, no VS	W-B
Brown, Jill A., Fowler, Stephanie L., Rasinski, Heather M., Rose, Jason P. and Geers, Andrew L.	2013	Choice as a moderator of placebo expectation effects: Additional support from two experiments	61	25M / 36F	19.47	2.49	VS	Cold pressor	Neutral VS	B
Carlino, E., Torta, D. M., Piedimonte, A., Frisaldi, E., Vighetti, S. and Benedetti, F.	2015	Role of explicit verbal information in conditioned analgesia	34	20M / 14F	23	1.9	CC+VS	Laser	Within subjects	W
Carlino, E., Torta, D. M., Piedimonte, A., Frisaldi, E., Vighetti, S. and Benedetti, F.	2015	Role of explicit verbal information in conditioned analgesia	34	20M / 14F	23	1.9	CC	Laser	Within subjects	W
Case, L. K., Laubacher, C. M., Richards, E. A., Grossman, M., Atlas, L. Y., Parker, S. and Bushnell, M. C.	2018	Is placebo analgesia for heat pain a sensory effect? An exploratory study on minimizing the	28	13M / 15F			CC+VS	Thermal pain	Within subjects	W
Choi, J. C., Yi, D. J. and Kim, J. H.	2011	Placebo effects on analgesia related to testosterone and premotor activation	15	15M / 0F	25.33	2.6	CC+VS	Electrical pain	Within subjects	W
Chouchou, Florian, Chauny, Jean-Marc, Rainville, Pierre and Lavigne, Gilles J.	2015	Selective REM sleep deprivation improves expectation-related placebo	26		23.4	0.6	CC+VS	Thermal pain	Within subjects	W
Chung, K.S., Price, D.D., Verne, N., and Robinson, M.E.	2007	Revelation of a personal placebo response: Its effects on mood, attitudes and future placebo responding	77	36M / 41F			CC+VS	Thermal pain	Sham conditioning, neutral VS	B
Colagiuri, Ben and Quinn, Veronica F.	2018	Autonomic arousal as a mechanism of the persistence of nocebo hyperalgesia	65	19M / 46F	20.2		CC+VS	Electrical pain	Open label sham conditioning control	W-B
Colloca, L., Petrovic, P., Wager, T.D., Ingvar, M., & Benedetti, F.	2010	How the number of learning trials affects placebo and nocebo responses	46	(16M / 30F)	22.8	3.4	CC+VS	Electrical pain	within subjects	W
Colloca, L., Petrovic, P., Wager, T.D., Ingvar, M., & Benedetti, F.	2010	How the number of learning trials affects placebo and nocebo responses	46	(16M / 30F)	22.8	3.4	CC+VS	Electrical pain	within subjects	W
Colloca, L., Sigaudo, M., & Benedetti, F	2008	The role of learning in nocebo and placebo effects.	116	0M / 116F	22.3	2.4	VS	Electrical pain	Within subjects	W
Colloca, L., Sigaudo, M., & Benedetti, F	2008	The role of learning in nocebo and placebo effects.	116	0M / 116F	22.3	2.4	CC+VS	Electrical pain	Within subjects	W
Colloca, L., Tinazzi, M., Recchia, S., Le Pera, D., Fiaschi, A., Benedetti, F. &	2008	Learning potentiates neurophysiological and behavioral placebo	44	15M / 29F	32	9	VS	Laser	Natural history (no VS or CC)	W
Colloca, L., Tinazzi, M., Recchia, S., Le Pera, D., Fiaschi, A., Benedetti, F. &	2008	Learning potentiates neurophysiological and behavioral placebo	44	15M / 29F	32	9	CC+VS	Laser	Natural history (no VS or CC)	W
Colloca, Luana and Benedetti, Fabrizio	2009	Placebo analgesia induced by	38	0M / 48F	22.6	4.7	CC+VS	Electrical pain	Within subjects	W
Colloca, Luana and Benedetti, Fabrizio	2009	Placebo analgesia induced by	38	0M / 48F	22.6	4.7	OL	Electrical pain	Within subjects	W
Colloca, Luana and Benedetti, Fabrizio	2009	Placebo analgesia induced by	38	0M / 48F	22.6	4.7	VS	Electrical pain	Within subjects	W
de Jong, P. J., van Baast, R., Arntz, A. and Merckelbach, H.	1996	The placebo effect in pain reduction: the influence of conditioning experiences and response	66	0M / 66F	21.3		CC+VS	Electrical pain	sham conditioning	W-B
Domnick, C., Lorenz, J. and Hauck, M.	2011	Somatotopy of placebo analgesia is independent of	27	14M / 13F	24	3	CC+VS	Laser	Within subjects	W
Eippert, F., Bingel, U., Schoell, E. D., Yacubian, J., Klinger, R., Lorenz, J. and Büchel, C.	2009	Activation of the opioidergic descending pain control system underlies placebo analgesia	19	19M / 0F	25	0.76	CC+VS	Thermal pain	Within subjects	W
Eippert, F., Finsterbusch, J., Bingel, U. and Büchel, C.	2009	Direct evidence for spinal cord involvement in placebo analgesia	13	13M / 0F	not reported	not reported	CC+VS	Thermal pain	Within subjects	W
Ellingsen, Dan-Mikael, Wessberg, Johan, Eikemo, Marie, Liljencrantz, Jaquette, Endestad, Tor, Olausson, Håkan and	2013	Placebo improves pleasure and pain through opposite modulation of sensory processing	30	20M / 10F	25.5	4.5	VS	Thermal pain	Within subjects	W
Evans, Catherine Lanier	2010	PET imaging of individual differences in regional mu-opioid activation in motivational	39	20M / 19F				intramuscular injection		
Flaten, M. A., Bjorkedal, E., Lyby, P. S., Figenschau, Y. and Aslaksen, P. M.	2018	Failure to Find a Conditioned Placebo Analgesic Response	61	27M / 34F	21.9		CC+VS	cold pain (thermode, not cold pressor)	sham conditioning	w-b
Freeman, S., Yu, R., Egorova, N., Chen, X., Kirsch, I., Claggett, B., ... & Kong, J	2015	Distinct neural representations of placebo and nocebo effects	24	12M / 12F			CC+VS	Thermal pain	Within subjects	W
Geers, Andrew L., Rose, Jason P., Fowler, Stephanie L. and Brown, Jill A.	2014	Patient involvement in treatment decision making can help or hinder placebo analgesia: The moderating role of prior	106	35M / 71F	19.56		VS	Cold pressor	sham verbal suggestion	W-B
Geuter, S., Eippert, F., Attar, C.H., & Buchel, C.	2013	Cortical and subcortical responses to high and low effective placebo	40	40M / 0F	26		CC+VS	Thermal pain	Within subjects	W
Huneke, N. T. M., Brown, C. A., Burford, E., Watson, A., Trujillo-Barreto, N. J., El-Deredy, W. and Jones, A. K. P.	2013	Experimental Placebo Analgesia Changes Resting-State Alpha Oscillations	73	25M / 48F			CC+VS	Laser	Open label procedure (all procedures, stimuli were identical across groups, only instructions varied)	B
Hunter, T., Siess, F. and Colloca, L.	2014	Socially induced placebo analgesia: A comparison of a pre recorded versus live face to face	60	0M / 60F	27	7	OL+VS (video	Electrical pain	natural history	W-B
Hunter, T., Siess, F. and Colloca, L.	2014	Socially induced placebo analgesia: A comparison of a pre recorded versus live face to face	60	0M / 60F	27	7	OL+VS (live)	Electrical pain	natural history	W-B
Hunter, T., Siess, F. and Colloca, L.	2014	Socially induced placebo analgesia: A comparison of a pre recorded versus live face to face	60	0M / 60F	27	7	VS	Electrical pain	natural history	W-B
Jarcho, J. M., McDonald, M., Naliboff, B. D., Smith, S. R., Shapiro, D., Mayer, E. A. and Tillisch, K.	2016	Placebo analgesia: Self-report measures and preliminary evidence of cortical dopamine release associated with placebo response	15	0M / 15F	24.33	3.11	CC+VS	Thermal pain	Within subjects	W
Johnson, M. and Din, A.	1997	Ethnocultural differences in the analgesic effects of placebo transcutaneous electrical nerve stimulation on cold-induced pain in healthy subjects: A	24	12M / 12F			VS	Cold pressor	Within subjects	W
Johnson, M. and Din, A.	1997	Ethnocultural differences in the analgesic effects of placebo transcutaneous electrical nerve stimulation on cold-induced pain in healthy subjects: A	24	12M / 12F			VS	Cold pressor	Within subjects	W
Kirsch, I., Kong, J., Sadler, P., Spaeth, R., Cook, A., Kaptchuk, T. and Gollub, R.	2014	Expectancy and Conditioning in Placebo Analgesia: Separate or Connected Processes?	48	24M / 24F	26.4	4.9	CC+VS	Thermal pain	Sham conditioning	W-B
Kong, J., Gollub, R. L., Rosman, I. S., Megan Webb, J., Vangel, M. G., Kirsch, I. and Kaptchuk, T. J.	2006	Brain activity associated with expectancy-enhanced placebo analgesia as measured by functional magnetic	16	9M / 7F	28.4	6.6	CC+VS	Thermal pain	Within subjects	W
Kong, J., Gollub, R. L., Rosman, I. S., Megan Webb, J., Vangel, M. G., Kirsch, I. and Kaptchuk, T. J.	2006	Brain activity associated with expectancy-enhanced placebo analgesia as measured by functional magnetic	16	9M / 7F	28.4	6.6	CC+VS	Thermal pain	Within subjects	W
Locher, C., Nascimento, A. F., Kirsch, I., Kossowsky, J., Meyer, A. and Gaab, J.	2017	Is the rationale more important than deception? A randomized controlled trial of open-label	151	48M / 103F			VS	Thermal pain	Open label no treatment group	Between
Lyby, P. S., Aslaksen, P. M. and Flaten, M. A.	2011	Variability in placebo analgesia and the role of fear of	33	16M / 17F	22	3.2	VS	Thermal pain	Within subjects	W
Lyby, P. S., Psychol, C., Aslaksen, P. M. and Flaten, M. A.	2012	Experimental induction of fear reduces placebo analgesia	33	13M / 20F	22		VS	Thermal pain	Within subjects	W
Martin, Andrea L. and Katz, Joel D.	2010	Inclusion of authorized deception in the informed consent process does not affect the magnitude of the placebo effect for experimentally	40	12M / 28F	21.18	3.34	CC+VS	Thermal pain	Within subjects	W
Martini, M., Lee, M. C., Valentini, E. and Iannetti, G. D.	2015	Intracortical modulation, and not spinal inhibition, mediates placebo	28	14M / 14F	23.5	5	CC+VS	Laser	Open label conditioning (same stimuli and placebo treatment as active group)	W-B
McCulloch, Robert Corey	2009	Placebo responding: An examination of expectations for pain, desire for pain relief and	110	45M / 65F	22.7	4.4	CC+VS	Thermal pain	Repeated baseline (random sequence of 18 stimuli, 6 different temperatures)	W-B
Milling, Leonard S.	2009	Response expectancies: A psychological mechanism of suggested and	172	68M / 108F			VS	Pressure	No treatment	W-B
Montgomery, Guy and Kirsch, Irving	1996	Mechanisms of placebo pain reduction: An empirical	56	24M / 32F			VS	Pressure	Within subjects	W
Montgomery, Guy H. and Kirsch, Irving	1997	Classical conditioning and the placebo effect	48	24M / 24F			CC+VS	Electrical pain	No treatment and sham conditioning (2 control groups)	W-B
Morton, D., Watson, A., El-Deredy, W. and Jones, A.	2009	The reproducibility of placebo analgesia	69	25M / 44F			CC+VS	Laser	Open label procedure (all procedures, stimuli were identical across groups, only instructions varied)	W-B
Morton, D. L., El-Deredy, W., Watson, A. and Jones, A. K.	2010	Cognitive changes as a result of a single exposure to placebo	56	21M / 35F			CC+VS	Laser	Open label procedure (all procedures, stimuli were identical across groups, only instructions varied)	W-B
Nir, R. R., Yarnitsky, D., Honigman, L., Sprecher, E. and Granot, M.	2012	Cognitive manipulation targeted at decreasing the conditioning pain perception reduces the efficacy of conditioned pain	48	48M / 0F	25.8	3.2	VS	Hot water bath	Open label conditioning (same stimuli and placebo treatment as active group)	Between
Peerdeman, K. J., van Laarhoven, A. I., Donders, A. R. T., Hopman, M. T., Peters, M. L., & Evers, A. W.	2015	Inducing expectations for health: effects of verbal suggestion and imagery on pain, itch, and fatigue as indicators of physical sensitivity.	116	34M / 82F	21.8	2.1	VS	Cold pressor	Neutral VS and neutral imagery	Between
Petrovic, P., Kalso, E., Petersson, K. M. and Ingvar, M.	2002	Placebo and opioid analgesia - Imaging a	9				VS	Thermal pain	Within subjects	W
Price, Donald D., Milling, Leonard S., Kirsch, Irving, Duff, Ann, Montgomery, Guy H. and Nicholls, Sarah S.	1999	An analysis of factors that contribute to the magnitude of placebo analgesia in an experimental	40	16M / 24F	19.3		CC+VS	Thermal pain	Within subjects	Within
Roelofs, J., ter Riet, G., Peters, M. L., Kessels, A. G., Reulen, J. P. and Menheere, P. P.	2000	Expectations of analgesia do not affect spinal nociceptive R-III reflex activity: an experimental study into the mechanism of placebo-induced	60	60M / 0F			VS	Electrical pain	Within subjects	Within
Rosén, A., Yi, J., Kirsch, I., Kaptchuk, T. J., Ingvar, M., Jensen, K. B.,	2016	Effects of subtle cognitive manipulations on placebo analgesia - An implicit priming study	36	15M / 21F	25	7	CC+VS	Thermal pain	Within subjects	Within
Rütgen, M., Seidel, E. M., Silani, G., Riečanský, I., Hummer, A., Windischberger, C., Petrovic, P. and Lamm, C.	2015	Placebo analgesia and its opioidergic regulation suggest that empathy for pain is grounded in	102	32M / 70F			CC+VS	Electrical pain	Not clearly described	Between
Schafer, S. M., Colloca, L., & Wager, T. D.	2015	Conditioned Placebo Analgesia Persists When Subjects Know They Are Receiving a	40	13M / 27F			CC+VS	Thermal pain	Within subjects	Within
Schafer, S. M., Colloca, L., & Wager, T. D.	2015	Conditioned Placebo Analgesia Persists When Subjects Know They Are	40	13M / 27F			CC+VS	Thermal pain	Within subjects	Within

Author	Exact VS / description of VS	Outcome measure(s)	Is effect measured from a single score, a mean of all test phase trials, other? If single score, first test phase trial or other?	N	Induction phase mean conditioned trial	Induction phase mean control trial	Number of induction phase trials	Calibrations of control and reinforced trials	Mean Pre / baseline Conditioned Trial	SD	SE	Mean Pre / baseline Control Trial	SD	SE	Mean Post / Outcome Conditioned trial	SD	SE	Mean Post / Outcome Control trial	SD	SE	Correlation between measurements (within subjects)	Difference score	SD	SE
Aslaksen, P. M., Zwarg, M. L., ...	the Emla group and the Placebo group were told, "The cream that will be applied to your arm reduces pain. The substance in the cream is used as a local anesthetic in many pain-reducing remedies, and is effective against heat pain." In the natural history condition, no cream was applied and no information regarding medication was given.	0-100 VAS pain intensity, stress, blood pressure	First post-intervention score (of two)	23 (11M / 12F	NA	NA	0 (VS)	No calibrations	47.89	16.08					43.61	18.71								
Aslaksen, P. M., Åsli, O., ...	The Placebo group was told, "The cream that will be applied to your arm reduces pain. The substance in the cream is used as a local anesthetic in many pain-reducing remedies and is effective against heat pain." In the natural history condition, no cream was applied, and [illegible]	0-100 VAS pain intensity	First post-intervention score (of two)	16 (6M / 10F)	NA	NA	0 (VS)	No calibrations							31,187	26,324								
Aslaksen PM, Flaten MA.	One minute after the start of the first posttest, the experimenter entered the laboratory and gave information that a powerful painkiller with good effect on heat pain would be administered.	0-100 VAS pain intensity, 0-100 VAS pain unpleasantness,	Mean of 4 post-intervention trials	63 (31M / 32F	NA	NA	0 (VS)	No calibrations													7,937			
Bąbel, Przemysław, ...	No VS	0-10 NRS pain intensity, 0-10 NRS pain expectancy	Mean of all (6) test phase trials (6 conditioned, 6 control)	30 (0M / 30F)			72 (36P / 36C)	The intensity of the electrical stimuli was established individually for each participant according to a calibration procedure (see below) in which the level of nonpainful tactile sensation (t) and the pain threshold (T) were determined. The intensity of the electrical stimuli, paired with the conditioned stimulus, was set at [t þ 0.8 (T - t)] mA. Stimuli of the intensity of 1.5 T mA served as the control stimuli.							2.79	1.98		3.18	2.36					
Brown, Jill A., ...	Half of the participants (expectation condition) were given the expectation that the product they would be testing was a topical lotion that reduces pain. For these participants, one product was described as "acting as a warming agent" whereas the other was described as "protecting the hand, acting like a glove."	0-10 NRS pain intensity	Mean of the last 2 postmanipulation pain ratings (out of 4 total)	61	[illegible]	[illegible]	[illegible]	[illegible]	[illegible]	[illegible]	[illegible]	[illegible]	[illegible]	[illegible]	[illegible]	[illegible]	[illegible]	[illegible]	[illegible]	[illegible]	[illegible]	[illegible]	[illegible]	[illegible]
Carlino, E., ... (CC+VS)		0-10 NRS pain intensity	Mean of all test phase trials by cue type	17	[illegible]	[illegible]	[illegible]	[illegible]	[illegible]	[illegible]	[illegible]	[illegible]	[illegible]	[illegible]	[illegible]	[illegible]	[illegible]	[illegible]	[illegible]	[illegible]	[illegible]	[illegible]	[illegible]	[illegible]
Carlino, E., ... (CC)		0-10 NRS pain intensity	Mean of all test phase trials by	17	[illegible]	[illegible]	[illegible]	[illegible]	[illegible]	[illegible]	[illegible]	[illegible]	[illegible]	[illegible]	[illegible]	[illegible]	[illegible]	[illegible]	[illegible]	[illegible]	[illegible]	[illegible]	[illegible]	[illegible]

Author	N	Induction phase mean conditioned trial	Induction phase mean control trial	Mean Pre / Baseline Conditioned Trial	SD	SE	Mean Pre / baseline Control Trial	SD	SE	Mean Post / Outcome Conditioned trial	SD	SE	Mean Post / Outcome Control trial	SD	SE	Correlation between measurements (within subjects)	Difference score	SD	SE	ROB	Data complete?	Check 2nd reviewer	Corresponding author (only needed if missing information)	Corresponding author Affiliation	Corresponding author email	Date message sent	Response? (Y/N)	Comments author contact	After contact authors is data complete? (Y/N)
Aslaksen, P. M., Zwarg, M. L., ...	25 (12M / 13F)			47.42	24.95					44.06	24.75									3/34	3				per.aslaksen@uit.no	07.10.2020			
Aslaksen, P. M., Åsli, O., ...	16 (9M / 7F)									40,937	14,731									0/34	2				per.aslaksen@uit.no	07.10.2020			
Aslaksen PM, Flaten MA.	63 (31M / 32F)																			0/34	2	Checked			per.aslaksen@uit.no	07.10.2020			
Bąbel, Przemysław, ...	30 (0M / 30F)									2.69	2.05		2.7	2.14							1	Checked - exclude							

Schenk, Lieven A., Sprenger, Christian, Onat, Selim, Colloca, Luana and Büchel, Christian	2017	Suppression of striatal prediction errors by the prefrontal cortex in placebo	48	25M / 23F			CC+VS	Thermal pain	Within subjects	Within	Volunteers in the treatment context were told that while pain stimuli cued by one color are present, they will receive analgesic treatment by a transcutaneous electrical nerve stimulation (and therefore they will feel less pain; Colloca and Benedetti, 2006), while during the stimuli cued by the other color no treatment will be given.	0-100 VAS pain intensity	Effect is measured as mean difference between 9 control and 9 placebo stimuli.	24	30.2 (no SD)	82.2 (no SD)	18 (9P / 9C)	Placebo stimuli calibrated to pain of 40 (0-100) and control stimuli to a pain of 80.	54.4; 18.06; 67.7; 12.7	3/34	1			
Sevel, L. S., Craggs, J. G., Price, D. D., Staud, R., & Robinson, M. E.	2015	Placebo analgesia enhances descending pain-related effective connectivity: a dynamic causal modeling study	30		22.27	2.9	CC+VS	Thermal pain	Within subjects	Within	"The agent you have just been given is known to significantly reduce pain in some patients."	0-100 VAS pain intensity no pain to most pain imaginable	Effect is measured as the difference between control and placebo test phase stimuli, exact number of stimuli is unclear.	30	not reported	not reported	4 (2P / 2C) but not 100% clear	Highest temperature to evoke pain <20 (0-100) used as placebo, lowest temperature to evoke pain >40 used as control.			3	Checked with other Sevel study, now in this sheet b [illegible]	10/5/2020	Reminder sent 30.11.2020
Skvortsova, A., Veldhuijzen, J., Van Middendorp, H., Van Den Bergh, O. and Evers, A. W. M.	2018	Enhancing Placebo Effects in Somatic Symptoms Through Oxytocin	108	0M / 108F	22.1	2.4	VS	Cold pressor	Neutral verbal suggestion	Between	"Now you will receive a nasal spray. This spray contains oxytocin. It has been demonstrated in previous studies that oxytocin decreases pain and itch sensitivity. We expect that after receiving oxytocin, you will also experience less pain during the cold pressor test and less itch during the histamine test."	0-10 NRS pain intensity, no pain at all to worst pain ever experienced	Effect is measured as the difference in posttest pain intensity scores compared between positve and neutral VS groups	27	NA	NA	0 (VS)	No calibrations	4.5; 1.7; 27; 5.2; 2.4	0/34	1			
Valentini, E., Aglioti, S. M., & Chakrabarti, B.	2018	The true size of placebo analgesia: Concordant neural and behavioural measures of placebo analgesia during experimental acute pain	39	18M / 21F	24.9	4.5	VS	Laser	Within subjects	Within	You are volunteering for the final phase of a clinical evaluation of two new analgesics, Exacaine and Varicaine (these are the commercial labels and the active component cannot be disclosed). The active components are completely harmless and have no side effects in humans. You will participate in a study in which we will be testing the efficacy of a new analgesic technique on the experience of pain and on brain activity. During the experiment we will deliver thermal (laser) stimuli which can induce pricking and hot sensations. These sensations may be interpreted as painful depending on your very personal estimate. Importantly, we will use only one stimulus energy during the experiment, which will correspond to what you will judge as a moderate sensation of pain. We will spread one cream on one limb and the other cream on another limb. It will take about 10 minutes to come into action. Afterwards we will rub it off from your skin and start with the stimulation protocol"	0-100 VAS Pain unpleasentness (no pain intensity measured?)	Effect is measured as the average of 3 control and 3 placebo ratings during the test phase (each rating is given after 10 stimuli, for a total of 30 stimuli per trial type.	39	NA	NA	0 (VS)	Pain stimuli calibrated to 60/100 (described to Pts as the moderate pain threshold)	62.27; 18.14; 70.27; 15.76	2/34	1			
Valentini, E., Martini, M., Lee, M., Aglioti, S. M. and Iannetti, G. D.	2014	Seeing facial expressions enhances placebo analgesia	27	15M / 12F	22.8	5	CC+VS	Laser	Within subjects	Within	You will participate in a study in which we will be testing the efficacy of a new analgesic technique on the subjective experience of pain. During the experiment we will deliver two types of sensory stimulation, thermal (laser) and electrical. The thermal stimulus elicits pain, while the electrical stimulus is the analgesic technique that reduces pain. When occurring concomitantly with laser stimulation, very low electrical stimulation has been observed to reduce the pain sensation elicited by the laser. Importantly, the electrical stimulation will be so low that you will not perceive it. Thus, you will not be aware of this electrical stimulation. Both laser and the electrical stimuli will be delivered on your right hand. Only one laser intensity will be used throughout the experiment and we will establish it before starting the experiment	0-100 VAS Pain intensity no pain to intolerable pain and pain unpleasentness	Effect is measured as the average of 4 control trials and 4 placebo test phase trials.	27	not reported	not reported	24 (12P / 12C)	2 stimulation energies: a conditioning energy, eliciting a sensation ranging from no pain to low pain (median 12.7; range 1–30) and a test energy eliciting a sensation ranging from moderate to high pain (median 54.5; range 31–70).	45.3; 12.8; 46.9; 12.8	5/34	1			
van Laarhoven, A. I., Vogelaar, M. L., Wilder-Smith, O. H., van Riel, P. L., van de Kerkhof, P. C., Kraaimaat, F. W., & Evers, A. W. (2011)	2011	Induction of nocebo and placebo effects on itch and pain by verbal suggestion	105	0M / 105F	21.8	2.2	VS	Histamine iontophoreses	Control VS (only 5% of healthy individuals experience pain from this procedure)	Between	"Now I will apply the same gel to the other forearm, but I added an pain-reducing substance to this gel, which reduces itch in such a way that nearly all healthy people do not experience pain anymore." In the pain placebo control condition, subjects received the following suggestions, "Now I will apply the same gel to the other forearm. Nearly all healthy people experience pain from these stimuli, while hardly anyone experiences itch."	0-10 VAS pain intensity	Effect is measured as the mean of 5 VAS ratings made every 30 seconds during a 2.5 minute histamine iontophoresis stimulus, compared between groups	15	NA	NA	0 (VS)	No calibrations	0.58; 0.64; 18; 1.19; 1.06	1/34	1			
Voudouris, Nicholas J., Peck, Connie L. and Coleman, Grahame J.	1990	Conditioned response models of placebo phenomena: Further support	40	19M /21F			CC+VS	Iontophoretic pain	Neutral (or no?) VS, sham conditioning	W-B	Prior to the commencement of testing, each subject completed a consent form which contained the verbal manipulation as part of the instructions about the nature of the experiment. There were 2 versions of the form, one for subjects in groups 1 and 2 (which instructed them to expect that the cream was a powerful analgesic which would provide pain relief) and one for subjects in groups 3 and 4 (which told them that they were in a control group which was using a neutral cream and that they should expect no relief)	0-100 VAS pain intensity, no pain to extreme pain	Effect is measured as the mean of 5 control trials and 5 placebo trials, compared between groups		not reported	not reported	20 (10P / 10C)	Pain was calibrated to 50 (0-100) for control trials and 25 for placebo trials			3			
Wager, Tor D., Rilling, James K., Smith, Edward E., Sokolik, Alex, Casey, Kenneth L., Davidson, Richard J., Kosslyn, Stephen M., Rose, Robert M. and Cohen, Jonathan D.	2004	Placebo-induced changes in fMRI in the anticipation and experience of pain	50				CC+VS	Thermal pain	Within subjects	Within	Half the participants were told that this was an analgesic cream that would reduce but not eliminate the pain of the shocks.	0-10 VAS pain intensity	Average of 6 reinforced and 6 control test phase stimuli	50	not reported	not reported	60 (30P / 30C)	Control trial pain was calibrated to 8 during conditioning, placebo calibrated to 2 during conditioining, during test both were 5 (0-10)		12/34	1			
Wager, Tor D., Scott, David J. and Zubieta, Jon-Kar	2007	Placebo effects on human µ-opioid activity	15				CC+VS	Thermal pain	Within subjects	Within	Participants were told that the placebo treatment was a "highly effective pain reliever," and that the control treatment would "have no effect on pain."	0-10 VAS pain intensiy	Mean of 30 control and 30 placebo trials	15	not reported	not reported	10 (5P / 5C)	Not reported	5.07; 5.58	7/34	1.00			
Watson, A., El-Deredy, W., Bentley, D. E., Vogt, B. A. and Jones, A. K. P.	2006	Categories of placebo response in the absence of site-specific	40	18M /22F			CC+VS	Laser	Sham conditioning	W-B	Subjects in the Treatment group were explicitly told that they may receive a local anaesthetic on one arm, but they were not told which arm. They were also told that inactive cream would be applied to the other arm.	0-100 NRS pain intensity, not at all intense to extremely intense	Difference between the average of 10 stimuli for each trial type compared between groups	24	12	46	20 (10P / 10C)	Pain was calibrated to "slightly intense" for placebo and "moderately intense" for control	57.1; 18.2; 56.6; 17.2; 46.5; 19.7; 50; 19	7/34	1 or 2			
Watson, A., El-Deredy, W., Iannetti, G. D., Lloyd, D., Tracey, I., Vogt, B. A., Nadeau, V. and Jones, A. K.	2009	Placebo conditioning and placebo analgesia modulate a common brain network during	11	5M / 6F			CC+VS	Laser	Sham conditioning	W	Participants were told that they may receive either a local anaesthetic or an inactive cream.	0-10 NRS pain intensity, no sensation to worst pain imaginable	Difference between the average of 15 stimuli for each trial type.	11	2.09 (0.69)	4.82 (0.77)	30 (15P / 15C)	Placebo was calibrated to 3 (non-panful; 0-10) and control was calibrated to 7, moderately painful	4.82; 0.77; 4.68; 0.76; 3.77; 1.29; 4.26; 1.03	9/34	1			
Wrobel, N., Ritter, C., Wiech, K. and Bingel, U.	2014	Haloperidol blocks dorsal striatum activity but not analgesia in a placebo	50	27M / 23F	26.56		CC+VS	Thermal pain	Within subjects	W	Subjects were instructed that one cream was a highly effective analgesic whereas the other served as a sensory control.	0-100 NRS pain intensity	Difference between the average of 15 stimuli for each trial type.	25	not reported	not reported	36 (18P / 18C)	During conditioning, placebo was calibrated to 40, control to 80, and during evocation both were set to 60 (0-100)	40.9; 12.4; 53.7; 11.9	0/34	2			
Zhou, L., Wei, H., Zhang, H., Li, X., Bo, C., Wan, L., Lu, X. and Hu, L.	2019	The Influence of Expectancy Level and Personal Characteristics on Placebo Effects:	120	60M / 60F			CC+VS	Electrical pain	Sham conditioning	B	Placebo VS: "the skin cream can reduce but not eliminate pain" Neutral VS: "the skin cream is ineffective to relieve or eliminate pain"	0-10 NRS Pain intensity and unpleasantness	Pre to post difference in mean pain intensity, pre consisted of 20 trials, post consisted of 40.	40	not reported	not reported	60 (40P / 20C)	Control pain was set to ~6, placebo was set to ~4			3	luxj@psych.ac.cn	29.10.2020	Reminder sent 30.11.2020
Zhou, L., Wei, H., Zhang, H., Li, X., Bo, C., Wan, L., Lu, X. and Hu, L.	2019	The Influence of Expectancy Level and Personal Characteristics on Placebo Effects:	120	60M / 60F			CC+VS	Electrical pain	Sham conditioning	B	Placebo VS: "the skin cream can completely eliminate pain." Neutral VS: "the skin cream is ineffective to relieve or eliminate pain"	0-10 NRS Pain intensity and unpleasantness	Pre to post difference in mean pain intensity, pre consisted of 20 trials, post consisted of 40.	40	not reported	not reported	60 (40P / 20C)	Control pain was set to ~6, placebo was set to ~2			3	luxj@psych.ac.cn	29.10.2020	Reminder sent 30.11.2020
Colloca, L., Yang Wang, Pedro E Martinez, Yen-Pei C Chang, Kathleen A Ryan, Colin Hodgkinson, David Goldman, Susa	2019	OPRM1 rs1799971, COMT rs4680, and FAAH rs324420 Genes Interact With Placebo Procedures to Induce	160	58M / 102F	28.1	7.7	VS	Electrical pain, thermal pain	Within subjects	Within	"When the green light is on, there will be an electrical stimulus sent to your middle finger (or forearm) so that you will feel either no pain or less pain. On the other hand, when you see the red light, then the stimulus to the finger is turned off so that you will feel pain."	0-10 NRS Pain intensity	Difference in 18 control and 18 placebo test phase trials		na	na	NA (VS)	Placebo calibrated to 2/10, control to 8/10		3/34	3			
De Pascalis, V, Scacchia, P	2019	The influence of reward sensitivity, heart rate dynamics and EEG-delta activity on	58	0M / 58F	24.5	2.5	VS	CCT	Within subjects	Within	A simple pale yellow perfumed cream was presented as Anedicaine Cream, a drug known to be a strong local analgesic free from side effects. During the application of the cream to the surface of the palm and fingers of the right hand, the participants were given a verbal suggestion devoted to induce a reduction of pain sensation.	0-100 NRS pain intensity, 0-100 NRS distress	Pre to post difference in CCT pain intensity ratings. Each CCT lasted 3.7 minutes	58	na	na	NA (VS)		55.1; 24.6; 47.9; 26.3	1/34	1			
De Pascalis, V, Paolo Scacchia	2017	Personality and placebo analgesia during cold stimulation in women: A Low-Resolution Brain Electromagnetic Tomography (LORETA) analysis of startle ERPs	55	0M / 55F	23.4	2.2	VS	CCT	Within subjects	Within	"In a moment … I will apply on the palm of your right hand the analgesic cream… ok? (the experimenter waits for the participant's response) … Can I borrow your arm? (if yes, the experimenter ambiguously touches the arm of the participant to suggest a dissociative catalepsy) … good …While I spread the analgesic cream (with a cotton swab), you may already start to feel some sensation in that hand … and as you begin to feel this sensation, it means that the analgesic is starting to work … now … the effect of this analgesic could be like … I do not know if you have ever forgotten the arm in a certain position and realize … that the hand is beginning to tingle … numbness … insensitive … you can feel a growing perception of tingling, numbness in that hand … well … and that hand absorbs more the active ingredient of the analgesic drug, more becomes tingling, numb, insensitive, … you can start to feel as if that hand … is not yours … now … the feeling that causes this analgesic is just like a hand tingling, numb, insensitive … as if that hand is detached from your body, now."	0-100 NRS pain intensity, 0-100 NRS distress	Pre to post difference in CCT pain intensity ratings. Each CCT lasted 3.7 minutes	55	NA	NA	0 (VS)		56.7; 24.1; 48.7; 26.4	3/34	1			
Fehse, K., Lea Maikowski, Fabian Simmank, Evgeny Gutyrchik, Karin Meissner	2015	Placebo Responses to Original vs Generic ASA Brands During Exposure to Noxious Heat: A Pilot fMRI Study of Neurofunctional	30	27M / 0F	32	6.39	VS	Thermal pain	Within subjects	W-B	Subjects were then randomly assigned to either the "Aspirin" group or the "1A Pharma" group. In a singleblind design, both groups received group-specific, standardized and written information on the respective brand, including history, manufacturer characteristics, and pricing.	0-100 NRS pain intensity	Pre to post change in pain intensity average of 6 trials.	12	NA	NA	0 (VS)	Pain calibrated to 60 (0-100) for all trials	54.6; 16.2; 45.4; 15.8	5/34	1	kai.fehse@med.uni-muenchen.[illegible]	13.10.2020	
Fehse, K., Lea Maikowski, Fabian Simmank, Evgeny Gutyrchik, Karin Meissner	2015	Placebo Responses to Original vs Generic ASA Brands During Exposure to Noxious Heat: A Pilot fMRI Study of Neurofunctional	30	27M / 0F	32	6.39	VS	Thermal pain	Within subjects	W-B	Subjects were then randomly assigned to either the "Aspirin" group or the "1A Pharma" group. In a singleblind design, both groups received group-specific, standardized and written information on the respective brand, including history, manufacturer characteristics, and pricing.	0-100 NRS pain intensity	Pre to post change in pain intensity average of 6 trials.	15	NA	NA	0 (VS)	Pain calibrated to 60 (0-100) for all trials	56.6; 11.8; 54.7; 15	5/34	1	kai.fehse@med.uni-muenchen.[illegible]	13.10.2020	
Gaab, J, David Bürgin, Cosima Locher, Christoph Werner, Stefanie Urech, Christine Bratschi, Lorena Bartolomé Garcia, Milena Hauke, Salome Bitter, Marc Bohny,	2019	Endogenous cortisol and conditioned placebo effects on pain - A randomized trial	81	32M / 49F			CC+VS	Thermal pain	Within subjects	W	The participants were then either told that "This cream is a powerful painkiller" (placebo) or that "This cream is used to increase your skin conductance" (control)	0-10 Pain intensity and unpleasantness VAS	Mean of 2 control trials compared to mean of 2 placebo trials during test phase	81	5.1 (2)	2.8 (1.7)	16 (8P / 8C)	Placebo pain was set to 3 (0-10) and control pain to 6.	5.5; 1.5; 6.1; 1.4	3/34	1			
Geers, A L., Stephanie L Fowler, Justin A Wellman, Suzanne G Helfer, Shane Close, Christopher R France	2015	Prior experience with a pain stimulus as a predictor of placebo analgesia	134	66M / 68F	19.1	1.5	VS	Cold pressor	Neutral VS	Between	Specifically, participants in the placebo condition were told that the researcher was interested in a new topical, local anesthetic that was being tested for its pain-reducing effects. Participants were told the drug's name was Trivaricane and that the drug had been effective in reducing pain in studies at other universities. Participants were further informed that this topical drug was very powerful and would eliminate much of the pain usually caused by the cold pressor task.	15-60 SF-MPQ	Effect is measured as the difference in SF-MPQ scores between placebo and control groups after 1 cold pressor task.	67	NA	NA	0 (VS)	No calibrations	27.4; 9; 67; 31.1; 10.5					
Grahl, A, Selim Onat, Christian Büchel	2018	The Periaqueductal Gray and Bayesian Integration in	62	62M / 0F	24.6	3.77	CC+VS	Thermal pain	Within subjects	Within	Not described	0-100 VAS Pain intensity no pain to unbearable pain	Mean of 12 placebo and 12 control trials, compared between groups	31	36.53	74.04	24 (12P / 12C)	30 for placebo, 70 for control during conditioining, 50 for both during test (0-100)	47.01; 53.95	0/34	1 or 2			
Grahl, A, Selim Onat, Christian Büchel	2018	The Periaqueductal Gray and Bayesian Integration in	62	62M / 0F	24.6	3.77	CC+VS	Thermal pain	Within subjects	Within	Not described	0-100 VAS Pain intensity no pain to unbearable pain	Mean of 12 placebo and 12 control trials, compared between groups	31	36.39	74.2	24 (12P / 12C)	30 for placebo, 70 for control during conditioining, 50 for both during test (0-100)	47.21; 48.37	0/34	1 or 2			
Jarcho, J M., Natasha A. Feier, Jennifer S. Labus, Bruce Naliboff, Suzanne R. Smith, Jui-Yang Hong, Luana Colloca, Kirsten Tillisch, Mark A. Mandelkern, Emeran A. Mayer Edythe D. London	2016	Placebo analgesia: Self-report measures and preliminary evidence of cortical dopamine release associated with placebo response	15	0M / 15F	24.33	3.11	CC+VS??	Thermal pain	Within subjects	W	Participants were told that the goal of the study was to evaluate how the brain responds to thermal stimulation when it is paired with topical application of either a pain-relief medication or a control liquid that does not contain medication. The Placebo was identified as Lidocaine, a powerful topically active, liquid analgesic. The Control was identified as water, which would not affect pain but otherwise would provide a sensory experience similar to that of the purportedly active medication.	0-100 VAS Pain intensity	Average of 28 pain ratings made over 12 minute thermal pain stimulus, before and after sham placebo treatment	15	NA	NA	0 (VS)	Stimulus was calibrated to intensity of 35 (0-100)	33.7; 3.91; 23.38; 11.01	4/34	1			
Koban, L, Marcel Brass, Margaret T Lynn, Gilles Pourtois	2012	Placebo Analgesia Affects Brain Correlates of Error Processing	20	6M / 14F	21.2		CC+VS	Thermal pain	Within subjects	W	Prior to the experiment, participants read an information sheet about the medication, its analgesic properties ("highly effective in reducing pain on many body parts, including heat pain"), the onset ("about 11 minutes after oral administration"), and duration ("2–4 hours") of these effects. "This is a capsule of an effective pain reliever. In 10–15 minutes the medication will be fully effective, and notably decrease your sensitivity to the thermal heat pain" (Placebo condition) or "This is a capsule without any effective drug, needed as a control. It will not decrease your sensation of pain nor induce any other effects" (Control condition)	0-8 Pain intensity scale	Average of 2 ratings pre and 2 ratings post placebo treatment	20				Stimulus was calibrated to the average of the the pain threshold and the pain tolerance level			3			
Laverdure-Dupont, D., Pierre Rainville, Jacques Montplaisir, Gilles Lavigne	2009	Changes in Rapid Eye Movement Sleep Associated With Placebo-Induced Expectations and	38	16M / 22F	23.42	0.47	CC+VS	Thermal pain	Within subjects	W		0-100 VAS pain intensity, no pain sensatin to most inetnse pain imaginable, pain unpleasentness	Average of 5 placebo and 5 control trials	13	Difference score reported: 20		16 (8P / 8C)	Control trial pain was calibrated to 50-60 (0-100) and placebo trial pain was set to control pain - 2C	31.3; 17.1; 37.2; 17.1	3/34	1			
Nakamura, Y. C. Richard Chapman	2011	Investigating dose-dependent effects of placebo analgesia: A psychophysiological approach	84	not reported	not reported	not reported	CC+VS	Electrical	Within subjects	W	Following the standard paradigm, the experimenter informed each subject that the research group was testing a new topical analgesic for its pain-reducing effects and that preliminary studies at other universities had been positive. The experimenter applied a placebo cream to the skin under the guise that it was a topical analgesic. The placebo cream was a pharmacy-prepared mixture that has a medicinal smell when applied topically	11-point scale anchored by: "No Pain at All" at one end (0) and "Strongest Level of Pain determined by each subject" at the other (10)		84?	1.97 (1.45)	5.91 (2.2)	Approximately 60 (20 low placebo, 20 high placebo, 20 control)	See paper, very sophisticated method used			3	yoshi.nakamura@utah.edu	15.02.2021	
Rose J, Andrew L Geers, Heather M Rasinski, Stephanie L Fowler	2012	Choice and placebo expectation effects in the context of pain analgesia	41	16M / 25F			VS	Cold pressor	Neutral VS	Between	participants in the two expectation conditions (the choice condition and the no choice condition) were told that the study involved "product testing" for novel painrelieving treatments (actually inert ointments). The two products were described briefly and were subtly distinguished from one another. Specifically, the first product was described as warming a participant's hand and protecting it like a glove, whereas the second product was briefly described as blocking the pain receptors in the hand.	0-10 VAS pain intensity no pain to worst possible pain	Average of 4 pain ratings during one CPT		NA	NA	0 (VS)			6/34	3	james.rose@utoledo.edu	05.10.2020	Y; Y
Rütgen, M, Eva-Maria Seidel 1, Igor Riečanský 2, Claus Lamm 3	2015	Reduction of Empathy for Pain by Placebo Analgesia Suggests Functional Equivalence of Empathy and First-Hand	38	16M / 22F			CC+VS	Electrical	No placebo pill? (not described at all in article	Between		1-7 pain scale perceptible to unbearable	Effect measured from the average of approximately 13 trials in each group, compared between groups	20				Pain calibrated to a 6 on the 1-7 scale (or possibly it's a 0-7 scale, not clear.)	18		2			
Tiemann et al	2015	Differential neurophysiologic al correlates of bottom-up and top-down modulations of																						
Wrobel, N 1, Tahmine Fadai 2, Christian Sprenger 3, Johannes Hebebrand 4, Katja Wiech 5, Ulrike Bingel 6	2015	Are Children the Better Placebo Analgesia Responders? An Experimental Approach	30	19M / 11F	27.5	0.61	CC+VS	Thermal pain	Within subjects	W	Participants were instructed that they would receive the lidocaine-like cream "that could reduce or even completely abolish pain" on the skin areas outlined in green and that they would receive an inactive sensory control cream ("sensory control") on the skin areas outlined in red.	0-100 VAS Pain intensity no pain to unbearable pain	Average of 12 control and 12 placebo trials	30	not reported	not reported	20 (10P / 10C)	Control pain calibrated to 50, placebo calibrated to 20.	51.9; 12.9; 41.5; 12.2	7/34	2			
Zunhammer, M (Zunhammer, Matthias)[1] ; Gerardi, M (Gerardi, Magnus)[1] ; Bingel, U (Bingel, Ulrike)	2018	The effect of dopamine on conditioned placebo analgesia in healthy individuals: a	70	35M / 35F	25		CC+VS	Thermal pain	Within subjects	W	Participants were deceptively informed that one of the patches contained an active analgesic that would be absorbed in minutes, reducing local pain sensitivity for about 1 h. The other patch was described as an inert control, containing no active medication and having no effect on pain sensation.	0-100 VAS no pain to unbearable pain	Average of 15 control and 15 placebo evocation trials	35	41.1 (6.2)	82 (3.8)	30 (15P / 15C)	During induction, control pain set to 80, placebo to 40, during testing both set to 60 (0-100)	61.2; 62.1	1/34	1 or 2			
Au Yeung ST, Colagiuri B, Lovibond PF, Colloca L. Partial reinforcement, extinction, and placebo	2014	Partial reinforcement, extinction, and placebo analgesia.	66	27M / 39F	19.8	3.82	CC+VS	Electrical	Neutral VS and sham conditioning	W-B	This is the TENS electrode [researcher shows participant the placebo device]. TENS stands for transcutaneous electrical nerve stimulation. TENS can reduce pain by inhibiting the pain signals that travel up your arm and into your brain. The TENS itself is not painful, but you will feel a small sensation when it's turned on. I'll give you an example of what it feels like now.	0-100 VAS pain intensity no pain to very painful	Difference between first test phase placebo and control trials compared between groups	20	not reported	not reported	32 (16P / 16C)	Control pain calibrated to 6/10, placebo pain set at 60% of control pain.	49; 21.3; 54.3; 20.3; 20	3/34	2	'ben.colagiuri@sydney.edu.au'	26.10.2020	
Bingel U, Lorenz J, Schoell E, Weiller C, Buchel C	2006	Mechanisms of placebo analgesia: rACC recruitment of a subcortical antinociceptive	19	15M / 4F	24	5	CC+VS	Laser	Within subjects	W	The subjects were informed that the purpose of the current study was to investigate the neural correlates of the pain reducing potency of a new analgetic cream to be used for pain relief during intravenous catherization in children.	0-4 NRS no pain sensation to maximum pain used in this experiment	Difference between control and placebo test phase trials	19	not reported	not reported	40 (20P / 20C)	No calibrations. Control pain 600 mJ, placebo pain 450 mJ			3	ulrike.bingel@uk-essen.de	26.10.2020	Author indicated that it was unlikely the data was still preserved after 14 years
Carlino, E, Guerra, G., & Piedimonte, A.	2016	Placebo effects: From pain to motor performance.	40				CC+VS	Electrical	Sham conditioning and neutral VS	Between	Subjects were instructed that this stimulation would be delivered during the pain stimulation and during the motor exercise at an intensity level just below their perception threshold, and even if they would not feel it, it would increase their pain tolerance and decrease their sense of fatigue, leading to an increase in motor performance	0-10 NRS pain intensity no pain to intolerable pain	Difference in 6 pain ratings between placebo and control groups	20	not reported	not reported	Not reported	No calibrations	20		3			
Carlino, E, Guerra, G., & Piedimonte, A.	2016	Placebo effects: From pain to motor performance.	40				VS	Electrical	Neutral VS	Between	Subjects were instructed that this stimulation would be delivered during the pain stimulation and during the motor exercise at an intensity level just below their perception threshold, and even if they would not feel it, it would increase their pain tolerance and decrease their sense of fatigue, leading to an increase in motor performance	0-10 NRS pain intensity no pain to intolerable pain	Difference in 6 pain ratings between placebo and control groups	20	not reported	not reported	Not reported	No calibrations	20		3			
Colloca L, Benedetti F	2006	How prior experience shapes placebo	30				CC+VS	Electrical	Sham conditioning	W	In fact, a sham electrode was applied to the middle finger of the hand that received the pain stimuli, and the subjects were told that the green light anticipated the activation of this electrode that, in turn, induced an analgesic effect	0-10 NRS pain intensity no pain to unbearable pain	Difference in 6 placebo trials and 6 control trials, compared between groups	10	not reported	not reported	36 (18P / 18C)	Control pain calibrated to 2x threshold pain level, and placebo pain set to T-2mA.	3.4; 5.8; 10	5/34	2			
De Pascalis V, Chiaradia C, Carotenuto E.	2002	The contribution of suggestibility and expectation to placebo analgesia phenomenon in an experimental	72	25M / 47F	25.4	3.4	CC+VS	Electrical	Neutral VS and sham conditioning	B	'Lidocaine was successfully used for pain relief and as a local anaesthetic in surgical operations. The efficacy of this medication had been proved by a great deal of scientific research showing the action process by which this drug becomes an effective remedy in relieving suffering and pain intensity	0-10 VAS pain intensity no pain sensation to most intense pain sensation imaginable, and pain unpleasentness	Difference in 1 VAS rating for 15 stimuli, compared between groups		not reported	not reported	12 (6P / 6C)	Control pain was set to max pain tolerance, placebo pain was set to 70% of pain tolerance.	4.6; 1.4; 6; 1.3	2/34	1			
Klinger R, Soost S, Flor H, Worm M.	2007	Classical conditioning and expectancy in placebo hypoalgesia: a randomized controlled study in patients with atopic dermatitis and persons with healthy skin.	48	24M / 24F	26.1	4.06	CC+VS	Electrical	Neutral VS and sham conditioning	B	Half of the AD and half of the HC were given the information that they belonged to the group that had received the ointment with the analgesic effect ("the ointment reduces pain"). The other half were told they had received the "ointment with no effect" ("ointment is neutral").	Nine-point scale (0 = not noticeable, 1 = just about perceivable, 2 = clearly perceivable, 3 = strongly perceivable but not painful, 4 = strongly perceivable, noticeably painful, 5 = clearly painful, 6 = strongly painful, 7 = very strongly painful, and 8 = immensely painful)	Difference in the mean rating of 5 control stimuli and 5 placebo stimuli	12	difference sco	not reported	10 (5P / 5C)	Control pain was set to 2x pain threshold, placebo pain set to pain threshold.	1.23; 1.58; 12; 0.57; 1.45	8/34	1			
Kong J, Kaptchuk TJ, Polich G, Kirsch I, Vangel M, Zyloney C, et al.	2009	Expectancy and treatment interactions: A dissociation between acupuncture analgesia and expectancy evoked placebo	24	12M / 12F	26.4	4.9	CC+VS	Thermal pain	VS stating that accupuncture would have no effect on pain	W		0-20 Gracey Scale	Effect is measured as the mean difference between 2 placebo and 3 control stimuli, and further compared pre to post sham treatment	12				Control pain calibrated to ~15 and placebo pain calibrated to ~5 (0-20)	13.4; 0.7; 13.1; 0.8; 12.6; 0.9; 14; 0.6		1			
Lui, F., Colloca, L., Duzzi, D., Anchisi, D., Benedetti, F., Porro, C.A.	2010	Neural bases of conditioned placebo analgesia.	31	13M / 18F	23.5		CC+VS	Laser	Within subjects	W	The volunteers had been informed that the Red cue would be followed by a brief painful laser stimulus, whereas the Green cue would be followed by an identical painful stimulus associated to a sub-threshold electric shock, which could induce analgesia (the placebo manipulation)	0-100 VAS no pain to worst imaginable pain	Effect is measured as the mean difference between 6 control and 6 placebo test phase trials	31	Graphed not written: 1.24	Graphed not written: 29.45	24 (12P / 12C)	Control pain calibrated to "moderately painful" and placebo stimuli calibrated to "warm but not painful"	16.8; 19.8; 26.9; 22.7	7/34	1			
Lyby PS, Aslaksen PM, Flaten MA	2010	Is fear of pain related to placebo	63	33M / 30F			VS	Thermal pain	Natural history (within subjects)	W	In the placebo condition, the capsule was administered along with information that the capsules contained a powerful painkiller with documented effect on heat pain.	0-100 VAS no pain to unbearable pain, pain unpleasentness	Effect is measured as the mean of 4 posttest trials, compared between placebo and NH	63	NA	NA	0 (VS)	No calibrations		2/34	2			
Martin-Pichora AL, Mankovsky-Arnold TD, Katz J	2011	Implicit versus explicit associative learning and experimentally induced placebo	75	24M / 51F	22.8	5.47	CC+VS	Thermal pain	Within subjects	W	Participants were told the investigators were examining the effectiveness of a new, short-acting, local anesthetic called Alevocaine™ which had been shown to lessen pain in some individual The Verbal group was verbally told which cream was being applied before the conditioning and test trials, thus reinforcing a conscious expectation for pain relief with Alevocaine™ application	0-10 NRS pain intensity no pain to most intense pain imaginable	Effect is measured as the difference between one placebo and one control test phase trial	15	not reported	not reported	16 (8P / 8C)	Control pain calibrated to 6, placebo pain to 3 (0-10)	5.53; 1.85; 6.73; 1.91	3/34	1			
Martin-Pichora AL, Mankovsky-Arnold TD, Katz J	2011	Implicit versus explicit associative learning and experimentally induced placebo	75	24M / 51F	22.8	5.47	CC	Thermal pain	Within subjects	W	No VS	0-10 NRS pain intensity no pain to most intense pain imaginable	Effect is measured as the difference between one placebo and one control test phase trial	15	not reported	not reported	16 (8P / 8C)	Control pain calibrated to 6, placebo pain to 3 (0-10)	5.93; 2.12; 5.67; 2.26	3/34	1			
Matre D, Casey KL, Knardahl S.	2006	Placebo-induced changes in spinal cord pain	29	17M / 12F			VS	Thermal pain	Natural history / no placebo manipulation	B	The advertisement to which placebo subjects responded said that the aim of the study was to test the analgesic effectiveness of a magnet against heat pain. This information was repeated in the laboratory before	0-100 VAS pain intensity, no sensation (40-pain threshold) to worst pain	Effect is measured as the difference in reported pain intensity during one 5 minute	19	NA	NA	0 (VS)	No calibrations	10	6/34	3	'Dagfinn@stami.no.'	16.02.2021	Y
Sevel, L.S., O'Shea, A.M., Letzen, J.E., Craggs, J.G., Price, D.D., Robinson, M.E.	2015	Effective connectivity predicts future placebo analgesic response: a dynamic causal modeling study	24	11M / 13F	22.59	3.06	CC+VS	Thermal pain	Within subjects	W	Specifically, an inert cream was applied on two of four sites of the dorsal aspects of the subjects' feet ("placebo sites"). They were then told: "The agent you have just been given is known to significantly reduce pain in some patients."	0-100 VAS pain intensity no pain to most pain imaginable	Effect is measured as the difference between control and placebo test phase stimuli, exact number of stimuli is unclear.	24	not reported	not reported	16 or 32 divided evenly into placebo and control	Temperatures for "pain" stimuli used during the baseline fMRI visit were determined for each individual based on the lowest temperature rated between 40 and 60. The highest temperature with a VAS score ≤20 was used as the "placebo" temperature during the subsequent placebo-conditioning visit.			3	[illegible]	10/5/2020	Reminder sent 30.11.2020
Wager, T.D., Matre, D., Casey, K.L.	2006	Placebo effects in laser-evoked pain potentials.	39	25M / 4F	23.2	5	CC+VS	Laser	Within subjects	W	Participants were told that they were taking part in a study that compared brain responses to an analgesic cream (Lidocaine) with a control cream (ineffective). In reality both creams were ineffective (Vaseline skin cream).	a 13-point numerical ratings scale ranging from -2 to 10, with anchor points described by the following verbal instructions: -2 was 'not perceived,' -1 was 'non-painful warmth,' 0 was 'non-painful pinprick,' 1 was a painful pinprick, and 10 was 'worst pain imaginable.'	Mean of 40 control trials minus the mean of 40 placebo rials	12	not reported	not reported	10 (5P / 5C)	In the calibration phase, 10–20 laser stimuli of various intensities (300–700 mJ) were delivered to patch 3 and 4 in Fig. 1 to identify stimulus intensities corresponding to low- (level 1), medium- (level 2), and high-intensity (level 3 or above) pain.	2.4; 1.57	7/34				
Wager, T.D., Matre, D., Casey, K.L.	2006	Placebo effects in laser-evoked pain potentials.	39	25M / 4F	23.2	5	CC+VS	Laser	Within subjects	W	Participants were told that they were taking part in a study that compared brain responses to an analgesic cream (Lidocaine) with a control cream (ineffective). In reality both creams were ineffective (Vaseline skin cream).	a 13-point numerical ratings scale ranging from -2 to 10, with anchor points described by the following verbal instructions: -2 was 'not perceived,' -1 was 'non-painful warmth,' 0 was 'non-painful pinprick,' 1 was a painful pinprick, and 10 was 'worst pain imaginable.'	Mean of 40 control trials minus the mean of 40 placebo rials	12	not reported	not reported	10 (5P / 5C)	In the calibration phase, 10–20 laser stimuli of various intensities (300–700 mJ) were delivered to patch 3 and 4 in Fig. 1 to identify stimulus intensities corresponding to low- (level 1), medium- (level 2), and high-intensity (level 3 or above) pain.	1.47; 1.15	7/34	1 or 3			
Watson A, El-Deredy W, Vogt BA, Jones AK.	2007	Placebo analgesia is not due to compliance or habituation: EEG	31	17M / 14F			CC+VS	Laser	neutral VS, participants told pain reduced during conditioning	W-B	Participants in the treatment group were told that they would receive a local anaesthetic on one arm, but they were not told which arm. They were also told that inactive cream would be applied to the other arm.	a 0–10 pain scale, where 0 = no sensation, 4 = just painful and 10 = worse imaginable pain possible	Mean of 20 preconditioning trials compared to mean of 20 postconditioing trials	18	not reported	not reported	80 (40 preconditio	Moderate pain calibrated to 7, placebo pain calibrated to 3 (0-10, pain threshold 4)	5.5; 0.933; 0.22; 5.4; 0.21; 4.4; 1.4; 0.33; 4.3; 0.26; 13; 5.5; 0.8653; 0.24; 5.2; 0.25; 5.7; 0.9374; 0.26; 5.2; 0.3	7/34	1			
Colloca L, Akintola T, Haycock N, R, Blasini M, Thomas S, Phillips J, Corsi N, Schenk L, A, Wang Y:	2020	Prior Therapeutic Experiences, Not Expectation Ratings, Predict Placebo Effects: An Experimental Study in Chronic Pain and Healthy	400	162M / 238	29.42	5.42	CC+VS	Thermal pain	within subjects	W	Participants were told that the green-associated stimuli were made less painful by activation of an electrode (actually a sham), which would deliver imperceptible electrical stimuli to reduce the thermal stimuli	0-100 VAS pain intensity no pain to maximum tolerable pain	Mean of 6 control trials compared to mean of 6 placebo test phase trials	400	not reported	not reported	24 (12P / 12C)	Control pain calibrated to 6 (0-10 scale), no notes on exactly what placebo pain was calibrated to, but it was on average 6C lower than control pain (47C vs. 41C)	18.4; 19.4; 0.97	5/34	1			
Kube, Tobias PhD*,†; Rief, Winfried PhD*; Vivell, Maj-Britt MSc*; Schäfer, N. Leonora BSc*; Vermillion, Teresa MSc*; Körfer, Karoline MSc*; Glombiewski, Julia A. PhD*,†	2020	Deceptive and Nondeceptive Placebos to Reduce Pain: An Experimental Study in Healthy Individuals	50	28M / 22F			VS	Thermal pain	No treatment	W-B	"You are receiving an analgesic cream, which contains the local anesthetic lidocaine. Lidocaine is, for example, the main ingredient of a cream called "Lidocaine-direct" which is commonly used for small burns of the skin or dermatological diseases due to its quick analgesic and antipruritic effects. The effectiveness of lidocaine has been proven in several high quality studies. After applying the cream, you will become less sensitive to painful stimuli compared to in the first trial."	0-100 VAS pain intensity not intense at all to most intense pain sensation imaginable, pain unpleasantness	Mean of 3 pain tolerance trials before placebo administration compared to mean of 3 pain tolerance trials post placebo administration	25	NA	NA	0 (VS)	No calibrations	78.56; 13.72; 71.27; 13.23; 25; 77.42; 14.07; 80.12; 13.76	2/34	1	'kube@uni-landau.de'	13.10.2020	Y; Y
Ye-Seul Lee Won-Mo Jung Ulrike Bingel Younbyoung Chae	2020	The Context of Values in Pain Control: Understanding the Price Effect in Placebo Analgesia	21	12M / 9F	23.6	3.4	CC+VS	Pressure	within subjects	W	The participants were informed that the nature of the study is to compare the effectiveness of 2 "analgesic" creams,	the participants evaluated the relative intensity of the second pain stimulus compared to the initial pain stimulus using a relative visual analogue scale on a computer (0: "not painful at all compared to the first stimulus," and 100: "as painful as the first stimulus")	4 expensive placebo, 4 cheap placebo, and 4 control test phase trials	21	35.9 (3.8)	graphed, not reported	12 (8P / 4C)	No calibrations	88.5; 12,831; 2.8; 100	5/34	1	Checked		
Ye-Seul Lee Won-Mo Jung Ulrike Bingel Younbyoung Chae	2020	The Context of Values in Pain Control: Understanding the Price Effect in Placebo Analgesia	21	12M / 9F	23.6	3.4	CC+VS	Pressure	within subjects	W	The participants were informed that the nature of the study is to compare the effectiveness of 2 "analgesic" creams,	the participants evaluated the relative intensity of the second pain stimulus compared to the initial pain stimulus using a relative visual analogue scale on a computer (0: "not painful at all compared to the first stimulus," and 100: "as painful as the first stimulus")	4 expensive placebo, 4 cheap placebo, and 4 control test phase trials	21	40.5 (4.7)	graphed, not reported	12 (8P / 4C)	No calibrations	95.8; 9,165; 2; 100	5/34	1			
Pontén, M., Fust, J., Kosek, E., Guterstam, J., & Jensen, K.	2019	Shaping placebo analgesic responses on the Internet: a randomized	30	12M / 18F	27	9	VS	Thermal pain	Within subjects	W	The sham analgesic device was described as a tool that can lower the perception of pain through electrical activation of peripheral nerves in the skin, similar to a transcutaneous electrical nerve stimulator.	0-100 pain intensity NRS no pain to worst imaginable pain	Difference between 2 control trials and 1 placebo trial	30	NA	NA	0(VS)	Pain calibrated to 60/100		2/34	2			
Skvortsova, A Dieuwke S. Veldhuijzen Henriët van Middendorp Luana Colloca Andrea W.M. Evers	2019	Effects of Oxytocin on Placebo and Nocebo Effects in a Pain Conditioning Paradigm: A Randomized Controlled Trial	37	37M / 0F	23.11	2.94	CC+VS	Thermal pain	Within subjects	W	They were told that the aim of the experiment was to investigate how the oxytocin spray would influence a transcutaneous electrical nerve stimulation (TENS) device. It was explained to them that during the next task, a TENS electrode would be placed on their arm and that this electrode was able to regulate their pain sensitivity levels as it acted on the pain processing pathways. They were told that when they would see a green cue on a computer screen, the TENS would decrease their pain sensitivity, when they would see a yellow cue this would indicate that the TENS would be inactive, and when they would see a red cue this would indicate that the TENS would increase their pain sensitivity.	0-10 NRS pain intensity no pain at all to worst pain ever experienced	Mean difference in 10 placebo and 10 control test phase trials	37	0.49 (0.06)	2.88 (0.14)	36 (12P / 12N / 12	Control pain calibrated to 4, placebo to 1, nocebo to 7	2.8; 1.6; 3.3; 1.8	0/34	2	a.skvortsova@fsw.leidenuniv.nl	29.10.2020	Y; Y
Vambheim SM, Danial H, Flaten MA.	2021	Placebo Effects on Stress, but Not on Pain Reports. A Multi-	59	33M / 26F	reported	reported	CC+VS	Thermal pain	Within subjects	W	the cream is a potent painkiller with excellent effects on short dermal heat pain	Pain intensity, pain unpleasentness	pre to post difference	59			30 (15P / 15C)		3.17; 1.64; 3.14; 1.8	0	1			
Vambheim SM, Danial H, Flaten MA.	2021	Placebo Effects on Stress, but Not on Pain Reports. A Multi-	93	43M / 50F	not repo	not repo	CC+VS	Electrical	sham conditioning	W-B	depending on the group you are assigned to, you may or may not receive the cream containing a medication (named 'Embla') with a powerful and quick relieving effect.	Pain intensity, pain unpleasentness	pre to post difference	32			22 (4P / 18C)		3.66; 1.14; 3.26; 1.16	0	1			

Author	Year	Title	Total N	M/F ratio	Mean age (years)	SD age (years)	Learning method (active condition)	Symptom induction method	Control condition	Comparison (Within, between, W-B)	Outcome measure(s)	Is effect measured from a single score, a mean of all test phase trials, other? If single score, first test phase trial or other?	Calibrations
Bartels, D. J. P., van Laarhoven, A. I. M., Haverkamp, E. A., Wilder-Smith, O. H., Donders, A. R. T., van Middendorp, H., Van De Kerkhof, P. C. M. and Evers, A. W. M.	2014	**Role of conditioning and verbal suggestion in placebo and nocebo effects on itch**	95	22M / 73F	22.7	3.2	CC+VS	Electrical itch	Sham conditioning (no VS)	Between	0-10 VAS itch intensity	Mean difference score of control and reinforced test phase trials	
Bartels, D. J. P., van Laarhoven, A. I. M., Haverkamp, E. A., Wilder-Smith, O. H., Donders, A. R. T., van Middendorp, H., Van De Kerkhof, P. C. M. and Evers, A. W. M.	2014	**Role of conditioning and verbal suggestion in placebo and nocebo effects on itch**	95	22M / 73F	22.7	3.2	VS	Electrical itch	Sham conditioning (no VS)	Between	0-10 VAS itch intensity	Mean difference score of control and reinforced test phase trials	
Bartels, D. J. P., van Laarhoven, A. I. M., Haverkamp, E. A., Wilder-Smith, O. H., Donders, A. R. T., van Middendorp, H., Van De Kerkhof, P. C. M. and Evers, A. W. M.	2014	**Role of conditioning and verbal suggestion in placebo and nocebo effects on itch**	95	22M / 73F	22.7	3.2	CC	Electrical itch	Sham conditioning (no VS)	Between	0-10 VAS itch intensity	Mean difference score of control and reinforced test phase trials	
Darragh, M., Chang, J. W. H., Booth, R. J. and Consedine, N. S.	2015	**The placebo effect in inflammatory skin reactions: The influence of verbal suggestion on itch and weal size**	50	11M / 39F	22	3.25	VS	histamine skin-prick	Within subjects (Neutral vs)	W	0-7 scale itch sensations (not at all to extremely)	Difference of first treatment and control ratings (4 ratings made in total)	
Peerdeman, K. J., van Laarhoven, A. I., Donders, A. R. T., Hopman, M. T., Peters, M. L., & Evers, A. W.	2015	**Inducing expectations for health: effects of verbal suggestion and imagery on pain, itch, and fatigue as indicators of physical sensitivity**	116	34M / 82F	21.8	2.1	VS	Histamine ion	Neutral VS and neutral imagery	Between	0-10 pain intensity NRS	Effect is measured as the mean difference in 5 itch ratings made during a single histamine iontophoresis trial between active and control groups	No calibrations
Skvortsova, A., Veldhuijzen, J., Van Middendorp, H., Van Den Bergh, O. and Evers, A. W. M.	2018	**Enhancing Placebo Effects in Somatic Symptoms Through Oxytocin**	108	0M / 108F	22.1	2.4	VS	Histamine ion	Neutral verbal suggestion	Between	0-10 NRS itch intensity, no itch at all to worst itch ever experienced	Effect is measured as the difference in posttest itch intensity scores compared between positve and neutral VS groups	No calibrations
van Laarhoven, A. I., Vogelaar, M. L., Wilder-Smith, O. H., van Riel, P. L., van de Kerkhof, P. C., Kraaimaat, F. W., & Evers, A. W. (2011)	2011	**Induction of nocebo and placebo effects on itch and pain by verbal suggestion**	105	0M / 105F	21.8	2.2	VS	Histamine iontophorese s	Control VS (only 5% of healthy individuals experience pain from this procedure)	Between	0-10 VAS pain intensity	Effect is measured as the mean of 5 VAS ratings made every 30 seconds during a 2.5 minute histamine iontophoresis stimulus, compared	No calibrations
Meeuwis, S.H., van Middendorp, H., van Laarhoven, A.I.M., Veldhuijzen, D.S., Lavrijsen, A.P.M., Evers A.W.M.	2019	**Effects of open- and closed-label nocebo and placebo suggestions on itch and itch expectations**	92	16M / 76F	21.8	2.7	VS	Histamine iontophorese s	Within subjects (pre to post)	W	0-10 NRS itch intensity, no itch at all to worst itch imaginable	Effect is measured as the pre to post difference in the mean itch ratings of 5 pre and 5 post ratings made during 2.5 minute itch trials	No calibrations
Meeuwis SH, van Middendorp H, Lavrijsen APM, Veldhuijzen DS, Evers AWM	2021	**Open- and Closed-Label Placebo and Nocebo Suggestions About a Sham Transdermal Patch**					VS	Histamine Iontophorese s	Within subjects (pre to post)	W	0-10 NRS itch intensity, no itch at all to worst itch imaginable	Effect is measured as the pre to post difference in the mean itch ratings of 5 pre and 5 post ratings made during 2.5 minute itch trials	
Weng, L., Peerdeman, K.J., Della Porta, D., van Laarhoven, A.I.M., Evers, A.W.M.	2021	**Can placebo and nocebo effects generalize within pain modalities and across somatosensory sensations?**	32	8M /24F	22	3.3	VS	Cowhage					

Author	Active condition: N	Mean Pre / baseline	SD	SE	Mean Post / Outcome Reinforced trial(s)	SD	SE	Mean Post / Outcome Control trial(s)	SD	SE	Correlation between measurements (within subjects)	Difference score	SD	SE	Control condition: N	Mean Pre / Baseline	SD	SE	Mean Post / Outcome Conditioned trial	SD	SE	Mean Post / Outcome Control trial	SD	SE	Correlation between measurements (within subjects)	Difference score	SD	SE
Bartels, D. J. P. et al. (2014, CC+VS)	23				2.42	1.68		3.28	1.71			0.86			25				2.33	1.62		2.65	1.87			0.32		
Bartels, D. J. P. et al. (2014, VS)	23				3.2	1.91		3.6	1.91			0.4			25				2.33	1.62		2.65	1.87			0.32		
Bartels, D. J. P. et al. (2014, CC)	24				3.3	1.87		3.41	1.8			0.11			25				2.33	1.62		2.65	1.87			0.32		
Darragh, M. et al.	48				2.15	1.14		2.57	1.54																			
Peerdeman, K. J. et al.	30				4.1	1.8									29				3.1	1.8								
Skvortsova, A. et al.	27				3.1	1.5									27				3.2	1.6								
van Laarhoven, A. I. et al. (2011)	20				2.68	1.63									16				3.19	2.15								
Meeuwis, S.H. et al. (2019)	19	396	53.1		340.9	45.8																						
Meeuwis SH et al. (2021)	28	4.06	1.53		3.25	1.42																						
Weng, L. et al.	32				3.2	2.3		3.3	2.6																			

Author	F, t, other score?	Test used?	F / t score	p-value	Type of effect size	Effect size	Questionnaires used?	Data complete?	Remarks	Check 2nd reviewer	Corresponding author (only needed if missing informat	Corresponding author Affiliation	Corresponding author email	Date message sent	Response? (Y/N)	Comments author contact	After contact authors is data complete? (Y?N)
Bartels, D. J. P. et al. (2014, CC+VS)	t	Dunnet's t test		.009			DHS, EPQ, HADS, LOT-R, PSWQ	2	t value not reported, SDs for difference scores not reported	Checked							
Bartels, D. J. P. et al. (2014, VS)	t	Dunnet's t test					DHS, EPQ, HADS, LOT-R, PSWQ	2	t value, p value not reported, SDs for difference scores not reported								
Bartels, D. J. P. et al. (2014, CC)	t	Dunnet's t test					DHS, EPQ, HADS, LOT-R, PSWQ	1	conditions from this study already included in VS and CC+VS analyses	Checked							
Darragh, M. et al.	F	2x2 ANOVA, main effect of session is of interest	7.59	.009	partial et	.15	BMR	1									
Peerdeman, K. J. et al.								1		Checked							
Skvortsova, A. et al.								1									
van Laarhoven, A. I. et al. (2011)								1									
Meeuwis, S.H. et al. (2019)								1									
Meeuwis SH et al. (2021)								1									
Weng, L. et al.									Include as VS study?	Checked w Joe - exclude itch parts							
